# *SKA1* promotes oncogenic properties in oral dysplasia and oral squamous cell carcinoma, and augments resistance to radiotherapy

**DOI:** 10.1002/1878-0261.13780

**Published:** 2024-12-10

**Authors:** Alexander Michael Grandits, Barbara Andrea Reinoehl, Renate Wagner, Peter Kuess, Franziska Eckert, Anna Sophie Berghoff, Thorsten Fuereder, Rotraud Wieser

**Affiliations:** ^1^ Division of Oncology, Department of Medicine I Medical University of Vienna Austria; ^2^ Department of Radiation Oncology Medical University of Vienna Austria; ^3^ Ludwig Boltzmann Institute for Hematology and Oncology Medical University of Vienna Austria

**Keywords:** HNSCC, metaphase, mitosis, oral leukoplakia, radioresistance, senescence

## Abstract

Oral squamous cell carcinoma (OSCC) is a malignancy associated with high morbidity and mortality, yet treatment options are limited. In addition to genetic alterations, aberrant gene expression contributes to the pathology of malignant diseases. In the present study, we identified 629 genes consistently dysregulated between OSCC and normal oral mucosa across nine public gene expression datasets. Among them, mitosis‐related genes were significantly enriched, including spindle and kinetochore‐associated complex subunit 1 (*SKA1*), whose roles in OSCC had been studied only to a very limited extent. We show that *SKA1* promoted proliferation and colony formation in 2D and 3D, shortened the duration of metaphase, and increased the migration of OSCC cell lines. In addition, high *SKA1* expression enhanced radioresistance, a previously unknown effect of this gene, which was accompanied by a reduction of radiation‐induced senescence. *SKA1* was also upregulated in a subset of advanced oral premalignancies and promoted tumor‐relevant properties in a corresponding cell line. Gene expression patterns evoked by *SKA1* overexpression confirmed that this gene is able to advance properties required for both early and advanced stages of tumorigenesis. In summary, our data show that *SKA1* contributes to malignant progression in OSCC and may be a useful marker of radioresistance in this disease.

## Graphical abstract

The mitosis‐related gene SKA1 was among 629 genes consistently dysregulated in OSCC across nine public gene expression datasets. In OSCC cell lines, SKA1 promoted proliferation and migration, enhanced radioresistance, and reduced radiation‐induced senescence. Accordingly, SKA1 expression correlated negatively with survival in radiotherapy‐treated OSCC patients. SKA1 promoted tumor‐related properties also in oral dysplasia, but activated different oncogenic pathways than in OSCC.

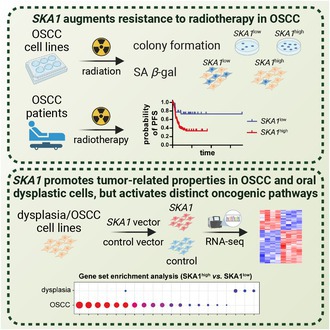

**Abbreviations**
DAPI: 4′,6‐Diamidino‐2‐phenylindol
DEG: differentially expressed gene
DMR: dose‐modifying ratio
DSMZ: Deutsche Sammlung von Mikroorganismen und Zellkulturen (German Collection of Microorganisms and Cell Cultures GmbH)
EMT: epithelial‐mesenchymal transition
FBS: fetal bovine serum
FC: fold change
FDR: false discovery rate
GA: gap area
GEO: Gene Expression Omnibus
GO: gene ontology
GSEA: gene set enrichment analysis
HNSCC: head and neck squamous cell carcinoma
HPV: human papillomavirus
OLP: oral leukoplakia
OSCC: oral squamous cell carcinoma
PFA: paraformaldehyde
qRT‐PCR: quantitative real‐time reverse transcriptase PCR
RNA‐seq: RNA‐sequencing
SA‐βgal: senescence‐associated β‐galactosidase
SEM: standard error of the mean
TCGA: The Cancer Genome Atlas

## 1 Introduction

Head and neck squamous cell carcinoma (HNSCC) is an umbrella term for cancers that arise from epithelial cells in the oral cavity, oropharynx, larynx, and hypopharynx. While these cancers exhibit distinct clinical, histological, and molecular characteristics [1], they are typically managed using similar treatment approaches [2]. Oral squamous cell carcinoma (OSCC) accounts for 30 to 50% of all HNSCC [3]. It is frequently linked to chronic consumption of tobacco and/or alcohol products [4, 5], but unlike other types of HNSCC, it is rarely associated with human papillomavirus (HPV) infection [4, 6]. In 2022, OSCC had an annual incidence of 4.2 and 4.0 per 100 000 inhabitants in Europe and worldwide, respectively [7]. Oral leukoplakia (OLP) is a commonly painless premalignant lesion that progresses to OSCC in 3% to >20% of cases [8, 9]. Optimal treatment of OSCC necessitates a multidisciplinary approach encompassing surgery, chemotherapy with agents such as cisplatin, 5‐fluorouracil or taxanes, and radiotherapy. The treatment is monomodal in early stages, and multimodal in advanced disease [2, 4]. Targeted therapies including the Epidermal Growth Factor Receptor antibody Cetuximab and the PD‐1 inhibitors pembrolizumab and nivolumab have so far not provided the anticipated benefits for patients with OSCC/HNSCC [2, 10, 11, 12].

The idea that a more profound comprehension of the biology of OSCC will facilitate the development of superior therapies has prompted the utilization of microarray‐ and RNA‐sequencing (RNA‐seq)‐based genome‐wide gene expression analyses across several cohorts including OSCC/HNSCC and corresponding normal tissue samples. These studies have been conducted in Europe, North America, Asia, and Australia and include patients with diverse ethnic and environmental backgrounds [1, 13, 14, 15, 16, 17, 18, 19, 20, 21, 22, 23]. They usually yield large numbers of differentially expressed genes (DEGs), which can be narrowed down to those more likely to contribute to tumor initiation and/or progression by comparing lists of DEGs from several independent datasets and by pathway enrichment analyses. Applying this approach to nine datasets including both OSCC and normal oral mucosa samples, we identified the Spindle And Kinetochore‐Associated Complex Subunit 1 (*SKA1*) gene to be significantly and consistently upregulated in OSCC. SKA1 was originally identified as a protein associated with the mitotic spindle and with kinetochores and required for timely progression from metaphase to anaphase [24, 25]. Together with spindle and kinetochore‐associated complex subunit 2 (SKA2) and spindle and kinetochore‐associated complex subunit 3 (SKA3), it forms the heterotrimeric SKA complex [26], which interacts with the quaternary NDC80 complex to strengthen the tethering of mitotic spindles to kinetochores [27]. Additional functions of SKA1 include the regulation of centrosome replication [28] and of gene transcription [29, 30].

Consistent with its roles in the cell cycle, *SKA1* also plays a role in malignant diseases. It was upregulated and/or associated with a poor prognosis in several tumor entities [28, 29, 30, 31, 32, 33, 34, 35, 36]. *SKA1* promoted proliferation, migration, and/or epithelial‐mesenchymal transition (EMT) in cell lines from various solid tumor types *in vitro* [30, 32, 34, 36, 37, 38, 39] as well as tumor growth and metastasis formation in mouse models [28, 30, 36]. Moreover, *SKA1* has been implicated in resistance to cisplatin in non‐small cell lung cancer [39] and to methotrexate in osteosarcoma [29]. However, the role of *SKA1* in OSCC and in the transformation of premalignant cells to outright cancer cells has been studied only to a very limited extent.

Our findings reveal that *SKA1* exhibited oncogenic properties in OSCC as well as in OLP cell lines and altered gene expression patterns in a manner that reflected these different disease stages. While *SKA1* did not affect the sensitivity of OSCC cells to chemotherapy, it did augment their resistance to irradiation and suppressed radiation‐induced senescence.

## 2 Materials and methods

### 2.1 Bioinformatics analyses

The Gene Expression Omnibus (GEO) database was searched using the term “OSCC OR “oral squamous cell carcinoma” OR HNSCC OR “head and neck cancer””. Seven independent microarray gene expression datasets were retrieved (Table S1). Optimal Affymetrix probe sets were selected from the jetset list [40]. Raw gene‐level counts of the RNA‐seq dataset GSE184616, also found through the GEO search, were retrieved from GREIN (http://www.ilincs.org/apps/grein/) [41]. TCGA data were downloaded from the Xena platform (https://xenabrowser.net/) [42]. For all datasets, non‐OSCC and, where information was available, HPV‐positive samples, were excluded from the analyses (Table S1). Genes dysregulated between OSCC and normal mucosa samples were identified using the r packages *limma* (microarrays) and *DESeq2* (RNA‐seq). For paired samples, paired tests were applied. The TCGA dataset was already downloaded as a list of DEGs that was generated on the Xena platform using an adapted version of the Maayan lab's Appyter bulk RNA‐seq analysis pipeline [42]. A false discovery rate (FDR) < 0.05 was considered statistically significant. Gene ontology (GO) analysis was performed on all genes that were significantly dysregulated in the same direction in all nine datasets using DAVID [43].

*SKA1* expression was correlated (Spearman's ρ) to the expression of all other genes in the six datasets with at least 50 OSCC samples (TCGA, GSE30784, GSE41613, GSE42743, GSE25099, GSE65858). *P* values were corrected for multiple testing using the Benjamini–Hochberg procedure. The list of genes that correlated to *SKA1* significantly (FDR < 0.05) and in the same direction in all datasets was termed the *SKA1* signature.

For correlation to clinical parameters and survival analyses, datasets GSE41613, GSE65858, and the TCGA data were used. *SKA1* expression was compared between groups defined by categorical variables using Student's *t*‐test (2 groups) or one‐way ANOVA (> 2 groups) and related to continuous variables through Spearman's ρ. For Kaplan–Meier analyses, optimal cutoffs for classification of patients into *SKA1* ^high^ and *SKA1* ^low^ groups were calculated using maximally selected rank statistics (r package *maxstat*). Statistical significance was evaluated through the log‐rank test, with adjustment for cutoff determination as described by Altman et al. [44]. Univariable Cox regressions were calculated for all available clinical parameters (r package *survival*). Significant parameters (*P* < 0.05) were included in the multivariable models.

To study the relation between *SKA1* expression and the degree of malignant progression from normal oral mucosa over dysplasia to OSCC, dataset GSE30784 was subjected to pseudotime analysis using the 8000 genes exhibiting the highest inter‐sample variability (r package *phenopath*).

### 2.2 Cell culture

The HPV‐negative human OSCC cell lines CAL‐33 (RRID:CVCL_1108) and SCC‐25 (RRID:CVCL_1682) were acquired from the German Collection of Microorganisms and Cell Cultures GmbH (DSMZ, Braunschweig, Germany). The human premalignant oral cell line MSK‐Leuk1 (RRID:CVCL_D684) had been established from a dysplastic, histopathologically atypical leukoplakia [45] and was purchased from the Memorial Sloan Kettering Cancer Center, New York City, NY, USA. CAL‐33 cells were cultivated in DMEM (Thermo Fisher Scientific, Waltham, MA, USA) containing 10% fetal bovine serum (FBS) (Thermo Fisher Scientific), 1× penicillin–streptomycin (Sigma‐Aldrich, St. Louis, MO, USA), and 2 mm L‐glutamine (Thermo Fisher Scientific). In 2D colony formation assays after fractionated irradiation of CAL‐33 derivative lines, FBS concentration was increased to 20%. SCC‐25 cells were cultured in DMEM/F12 (Thermo Fisher Scientific) supplemented with 20% FBS, 1× penicillin–streptomycin, 2 mm L‐glutamine, and 1 mm sodium pyruvate (Thermo Fisher Scientific). MSK‐Leuk1 cells were cultivated in keratinocyte SFM supplemented with 5 ng·mL^−1^ EGF and 30 μg·mL^−1^ pituitary extract or complete defined keratinocyte SFM (only for 2D colony formation) (all from Thermo Fisher Scientific). MSK‐Leuk1 cells were propagated on Primaria™ cell culture flasks and dishes (Corning, Corning, NY, USA). BHY (RRID:CVCL_1086), CAL‐27 (RRID:CVCL_1107), HN (RRID:CVCL_1283), and SCC‐4 (RRID:CVCL_1684) cells were acquired from the DSMZ and cultivated according to DSMZ recommendations. Phoenix GP cells (kindly provided by H. Stockinger, Medical University of Vienna, RRID:CVCL_H718) were cultivated in DMEM containing 10% FBS and 1× penicillin–streptomycin‐glutamine.

To induce the expression of shRNAs in SCC‐25 derivative cell lines, 1 μg·mL^−1^ doxycycline (MP Biomedicals, Santa Ana, CA, USA) was added to the medium 1 to 3 days prior to and during experiments.

All cells were cultivated in a cell culture incubator at 37 °C, 5% CO_2_, and 95% relative humidity. Cell lines were tested regularly for Mycoplasma contamination using the MycoAlert™ Mycoplasma Detection Kit (Lonza, Basel, Switzerland). The integrity and authenticity of the cell lines was ensured through rigorous monitoring of cell line characteristics, including morphological appearance, growth patterns, and cell doubling time. Moreover, the STR profiles (generated by Eurofins Genomics, Luxembourg City, Luxembourg) of the *SKA1* overexpression and knockdown cell lines corresponded to the profiles of the respective parent cell lines as reported by the DSMZ and Zhao et al. [46].

### 2.3 shRNA and overexpression constructs

Sequences of shRNAs targeting *SKA1* (shSKA1_1 and shSKA1_2) were obtained from [47]. To construct shSKA1_1 and shSKA1_2 expressing vectors, XhoI and EcoRI restriction sites were added to the respective 97‐mer oligonucleotides by PCR with primers 5′miR‐XhoI and 3′miR‐EcoRI. The resulting products were cloned into LT3REVIR using standard techniques. shRen.713 in LT3REVIR [47] was used as control (shCtrl).

For the generation of the *SKA1* overexpression vector, the *SKA1* coding sequence was amplified from cDNA of CAL‐33 cells by PCR with primers *SKA1*_outer_fwd and *SKA1*_outer_rev, followed by a nested PCR with primers *SKA1*_BamHI_fwd and *SKA1*_XhoI_rev. PCR products were cloned into pMSCV‐IRES‐*eGFP* using standard techniques. 97‐mers and primers were synthesized by Eurofins Genomics and their sequences are summarized in Table S2.

### 2.4 Viral transduction of cell lines

Phoenix GP cells were transfected with shRNA‐expressing lentiviral vectors along with helper plasmids pMD2.G and psPAX2 or with *SKA1*‐overexpression and empty retroviral vector along with helper plasmids pMD2.G and pGagPol using a standard calcium phosphate protocol. After 48 h, virus‐containing supernatant was collected, filtered through a 0.45 μm filter, and added to 50% confluent cells in the presence of 4 μg·mL^−1^ polybrene (Sigma‐Aldrich). Infection cycles were repeated after 24 h (lentiviral transduction) or after 24 and 48 h (retroviral transduction). Three days after the last cycle, cells were sorted for fluorescence marker positivity (Venus for shRNA constructs, eGFP for overexpression vectors) on a BD FACSAria™ Fusion cell sorter (BD Biosciences, Franklin Lakes, NJ, USA). Knockdown and overexpression of *SKA1* were confirmed by immunoblot analysis and quantitative real‐time reverse transcriptase PCR (qRT‐PCR).

### 2.5 RNA isolation, cDNA synthesis, and quantitative real‐time reverse transcriptase (qRT‐) PCR

Total RNA was isolated using TRIzol® reagent (Thermo Fisher Scientific), treated with DNAse I (Sigma‐Aldrich) according to the manufacturer's protocol, and reverse transcribed using the LunaScript™RT SuperMix Kit (NEB, Ipswich, MA, USA). GoTaq® qPCR master mix (Promega, Madison, WI, USA) and custom‐designed primers (Table S2) were used for qRT‐PCR on a StepOnePlus™ Real‐Time PCR instrument (Applied Biosystems™, Waltham, MA, USA) employing the instrument's standard cycling protocol for SYBR Green. Samples were measured in triplicate. Gene expression levels were normalized to those of *β*‐2‐microglobulin and to a reference sample using the ΔΔ*C*_T_ method [48].

### 2.6 Immunoblot analysis

Protein extracts were prepared using RIPA buffer [50 mm Tris/HCl pH 8.0, 0.1% SDS, 0.5% sodium desoxycholate (all from Sigma‐Aldrich), 150 mm NaCl (Carl Roth, Karlsruhe, Germany), 1% Triton X‐100 (Roche, Penzberg, Germany)] with 10% protease inhibitor (Sigma‐Aldrich). Protein concentrations were determined using the Bradford assay (Bio‐Rad Laboratories, Hercules, CA, USA). Samples were diluted to equal concentrations with 1× Roti®‐Load 1 (Carl Roth), and 20–40 μg of total protein were loaded per lane. SDS/PAGE was performed following standard protocols. Protein transfer to PVDF membranes (Pall, Port Washington, NY, USA) was conducted in a tank blotting unit overnight at 4 °C. Membranes were blocked with 4% bovine serum albumin (Sigma‐Aldrich) and 4% FBS in TBS‐T (40 mm Tris/HCl pH 7.6, 273 mm NaCl, 0.1% Tween® 20 (Sigma‐Aldrich)) for 1 h at room temperature. Incubation with primary antibodies (SKA1 (ab91550, Abcam, Cambridge, UK), cleaved caspase‐3 (Asp175) (#9664, Cell Signaling Technology, Cambridge, UK), LC3A/B (#12741, Cell Signaling Technology), CDKN1A (p21 Waf1/Cip1, #2947, Cell Signaling Technology), and GAPDH (#2118, Cell Signaling Technology); all at 1 : 1000) was performed in 1% bovine serum albumin and 1% FBS in TBS‐T overnight at 4 °C. The secondary antibody (goat‐anti‐rabbit‐horseradish peroxidase, 111055008, Jackson ImmunoResearch, West Grove, PA, USA; 1 : 10 000) was applied in TBS‐T for 1 h at room temperature. Immunoblots were developed with SuperSignal West Pico or Femto Chemiluminescent Substrates (Thermo Fisher Scientific), and signals were detected on a ChemiDoc Touch Imaging System (Bio‐Rad). Bands were quantified using image lab v6 (Bio‐Rad).

### 2.7 Cell proliferation assays

Real‐time cell proliferation assays were performed using the xCELLigence system (OMNI Life Science, Bremen, Germany). 2000 cells in 200 μL medium were seeded into wells of an RTCA E‐plate 16 (Agilent Technologies, Santa Clara, CA, USA). Cellular impedance was measured every 15 min for 120 h. Data were normalized to the 24 h time point. Linear mixed effects models were fitted to the data, and the significance of differences between the resulting curves was determined using *anova.lme* (r package *nlme* (v3.1.163)).

To test the proliferation of the cell lines under the conditions used for the scratch assay (chapter 2.11), the CellTiter‐Glo® Cell Viability Assay (Promega) was used according to the manufacturer's instructions.

### 2.8 2D and 3D colony formation assays

For 2D colony formation assays, 2000 (CAL‐33, SCC‐25) or 500 (MSK‐Leuk1) cells in 2 mL medium were seeded per well of a 6‐well plate and the medium was changed every 3–4 days. After 7–14 days, cells were fixed with methanol for 5 min and stained with 0.4% trypan blue (Merck, Darmstadt, Germany)/PBS for 20 min at room temperature. Wells were washed with PBS, dried, and photographed. Grayscale images were converted to binary images using the auto threshold function of imagej (National Institutes of Health, Bethesda, MD, USA), and colony numbers and sizes were quantified using fiji v1.54g (National Institutes of Health). Colonies in various stages of fusion were counted as one.

For 3D colony formation assays, 2000 (CAL‐33, SCC‐25) or 500 (MSK‐Leuk1) cells were suspended in 1 : 10 diluted Matrigel® (Corning) and seeded onto a layer of undiluted Matrigel®. 3D colonies were imaged after 3–5 days.

### 2.9 Cell death assay and cell cycle analysis

To assess the proportion of apoptotic cells, cells were trypsinized, washed once with ice‐cold PBS and once with ice‐cold Annexin V binding buffer (10 mm HEPES (Sigma‐Aldrich), 140 mm NaCl (Carl Roth), 2.5 mm CaCl_2_ (Carl Roth), pH 7.4), and stained with 1× Annexin V (BD Biosciences) and 1 μg·mL^−1^ 4′,6‐Diamidino‐2‐phenylindol (DAPI; BioLegend, San Diego, CA, USA) in Annexin V binding buffer for 15 min at room temperature. Cells were analyzed on an LSR Fortessa (BD Biosciences) flow cytometer.

For cell cycle analysis, cells were trypsinized and washed once with ice‐cold PBS and once with ice‐cold 2% FBS/PBS. Cell pellets were resuspended in 50 μL ice‐cold 2% FBS/PBS and fixed by adding 950 μL −20 °C cold 70% ethanol (Carl Roth). After > 30 min at −20 °C, cells were stained with 1 μg·mL^−1^ DAPI in ice‐cold 0.01% Triton‐X100/PBS. Cells were analyzed by flow cytometry on an LSR Fortessa flow cytometer, and cell cycle phases were assigned using flowjo v10.0.7r2. Cells with a DNA content higher than 4n (corresponding to the G_2_/M peak) were considered as hyperdiploid.

### 2.10 Proportions of cells in metaphase and metaphase duration

To assess the proportions of cells in metaphase, logarithmically growing cells were fixed with 4% paraformaldehyde (PFA, Sigma‐Aldrich)/PBS (Thermo Fisher Scientific) for 15 min, permeabilized with 0.1% Triton X‐100 (Fluka, Buchs, Switzerland)/PBS for 15 min, and stained with 1 μg·mL^−1^ DAPI/PBS for 5 min. All steps were performed at room temperature. Cells were washed once with PBS after each step. Images were taken on a BioTek Cytation 5 (Agilent Technologies) using a 20× objective. At least 200 non‐overlapping nuclei were counted per sample and categorized into interphase, metaphase, and anaphase.

For measurements of metaphase duration, 30 000 cells in 1 mL CO_2_‐independent medium (Thermo Fisher Scientific) were seeded per well of a 24‐well plate and incubated with 0.02 ng·mL^−1^ Hoechst 33342 (Thermo Fisher Scientific) in the cell culture incubator overnight. The sample chamber of the Cytation 5 was pre‐heated to 37 °C, plates were inserted, and cells were imaged with low exposure settings every 2.5 min for 10–16 h using a 4× objective (DAPI channel). Frames of all time points were aligned using fiji v1.54g and metaphase duration was evaluated manually for ≥ 25 randomly selected cells per sample that entered and exited metaphase during the experiment. The beginning and the end of metaphase were defined as the first frames in which the metaphase plate became visible as a prominent streak, and in which chromosome segregation could be observed as two parallel streaks, respectively.

### 2.11 Scratch assay and Transwell® migration assay

For the scratch assay, OSCC cell lines were grown to 95% confluence in wells of a 6‐well plate and then kept in medium with reduced FBS concentration that minimized proliferation (CAL‐33: 0.2% FBS; SCC‐25: 10% FBS [49]) for 1 day. MSK‐Leuk1 cells were prepared in a similar manner, but regular growth medium was used: the short duration of the assay was considered sufficient to rule out a relevant impact of proliferation on its results. Scratches were generated using a 200 μL pipette tip, and four representative positions were photographed per well at the beginning of the experiment (*t*_0_) and at defined time points thereafter (*t*_exptl_). Gap area (GA) was measured using fiji v1.54g and relative gap closure was calculated as follows: 1 – GA (*t*_exptl_)/GA (*t*_0_).

For the Transwell® migration assay, cells were kept in medium with reduced FBS concentration (CAL‐33: 0.2% FBS; SCC‐25: 10% FBS) for 1 day. 250 000 cells in 400 μL medium with reduced FBS were seeded into the Transwell® inserts (Corning). Inserts were placed into wells of a 24‐well plate with 700 μL normal growth medium in the lower chamber. After 24 h, cells attached to the membranes of the inserts were fixed with 4% PFA/PBS for 2 min, permeabilized with methanol for 20 min, and stained with 0.4% trypan blue/PBS for 20 min. All steps were performed at room temperature. Cells that had not migrated (thus remained attached to the top side of the insert) were removed using a cotton swab. Four representative positions of each insert were photographed and cells were counted manually.

### 2.12 Radiation treatment, radioresistance, DNA repair kinetics, and radiation‐induced senescence

Radioresistance was measured through 2D colony formation assays as described in chapter 2.8. CAL‐33, SCC‐25, and MSK‐Leuk1 derivative cell lines were seeded at 3000, 3000, and 700 cells, respectively, per well of a 6‐well plate and, starting on the day of seeding, exposed to total doses of 0–10 Gy of ionizing radiation (200 kV X‐ray; ~ 1 Gy·min^−1^, YXLON Maxishot, YXLON International GmbH, Hamburg, Germany) in five equal fractions with 24 h intervals [50]. Surviving fractions after irradiation were fitted using the linear quadratic model, and the dose‐modifying ratio (DMR) was calculated as described by Subiel et al. [51]. The significance of the deviations of the DMR ratios from 1 (indicating no effect) were tested using the ratio *t*‐test (r package *mratios*).

To measure radiation‐induced changes in cell size, cells were seeded into chamber slides (Nunc, Roskilde, Denmark), irradiated with 0 or 3 Gy on the day after seeding, and cultivated for 2 days. Cells were fixed with 4% PFA, permeabilized with 0.1% Tween‐20 (Sigma‐Aldrich) and 0.01% Triton X‐100, stained with Alexa Fluor™ 555‐Phalloidin (Thermo Fisher Scientific), and counterstained with 1 μg·mL^−1^ DAPI using standard procedures. Imaging was performed on a Cytation 5 using a 20× objective. Areas of ≥ 80 cells per sample were measured manually using fiji v1.54g.

Immunofluorescence analysis for γH2AX was performed on cells exposed to radiation doses of 2 or 1 Gy (CAL‐33 and SCC‐25 derivative cell lines, respectively) and allowed to recover for 0, 2, 4, 8, and 24 h. Cells were fixed with 4% PFA for 20 min, permeabilized with 0.2% Tween‐20 for 15 min, blocked with 2% bovine serum albumin (Sigma‐Aldrich) in PBS for 30 min, and stained with the anti‐γH2AX (Ser139) antibody (Cell Signaling, clone 20E3; 1 : 200) in 1% bovine serum albumin in PBS for 1 h at room temperature. Cover slips were mounted using ProLong™ Gold Antifade Mountant with DAPI (Thermo Fisher Scientific). Images were acquired on an Axio Imager M2 (Carl Zeiss AG, Oberkochen, Germany). γH2AX foci were counted manually.

For immunoblot analyses and senescence‐associated β‐galactosidase (SA‐βgal) assays, cells were exposed to a single dose of 0 (control), 3 (CAL‐33), or 5 Gy (SCC‐25, MSK‐Leuk1) of ionizing radiation. At the indicated times thereafter, proteins were harvested for immunoblot analyses. The Senescence β‐Galactosidase Staining Kit (Cell Signaling) was used 2–3 days after irradiation following the manufacturer's instructions. Color images were acquired on a Cytation 5 using a 20× objective.

### 2.13 RNA‐seq and data analysis

RNA was extracted from logarithmically growing cells using the RNeasy Mini Kit (QIAGEN, Venlo, The Netherlands). RNA‐seq was performed at the Core Facility Genomics, Medical University of Vienna. In brief, total RNA preparations were subjected to quality control on a Bioanalyzer 2100 (Agilent Technologies), sequencing libraries were prepared using the QuantSeq 3′ FWD protocol v2 (Lexogen, Vienna, Austria), and quality of libraries was checked on a Bioanalyzer 2100. Pooled libraries were sequenced on a P2 flow cell on a NextSeq2000 instrument (Illumina, San Diego, CA, USA) in 1 × 75 bp single‐end sequencing mode. Reads were demultiplexed (*idemux*), trimmed and filtered (*cutadapt* v2.8), aligned to the human reference genome (GrCh38 with Gencode 29 annotations), and counted (*star aligner* v2.61a). DEGs were identified using the r package *deseq2* v1.22.2 and applying an FDR of < 0.05 and subjected to Gene Set Enrichment Analysis (gsea v4.1) [52].

### 2.14 Statistics

At least three independent biological replicates were performed for each experiment. Data are presented as means ± SEM (standard error of the mean) of the mean values derived from each replicate. Student's two‐sided *t*‐test was used to compare the means of two independent groups. One‐way ANOVA followed by Dunnett's multiple comparison test was used for comparisons between multiple independent groups and a single control group, and two‐way ANOVA followed by Bonferroni's *post‐hoc* test was used for multiple groups with two or more factors. If the control sample had no variance due to normalization, a one‐sample *t*‐test was performed, and *P* values were corrected for multiple testing through the Bonferroni method where applicable.

Statistical tests were performed using graphpad prism 6 software (GraphPad Software, San Diego, CA, USA) and r v4.1.0. *P* values < 0.05 or, where applicable, corrected *P* values and FDRs < 0.05 were considered statistically significant.

## 3 Results

### 3.1 Identification of genes recurrently dysregulated in OSCC

**Fig. 1:**
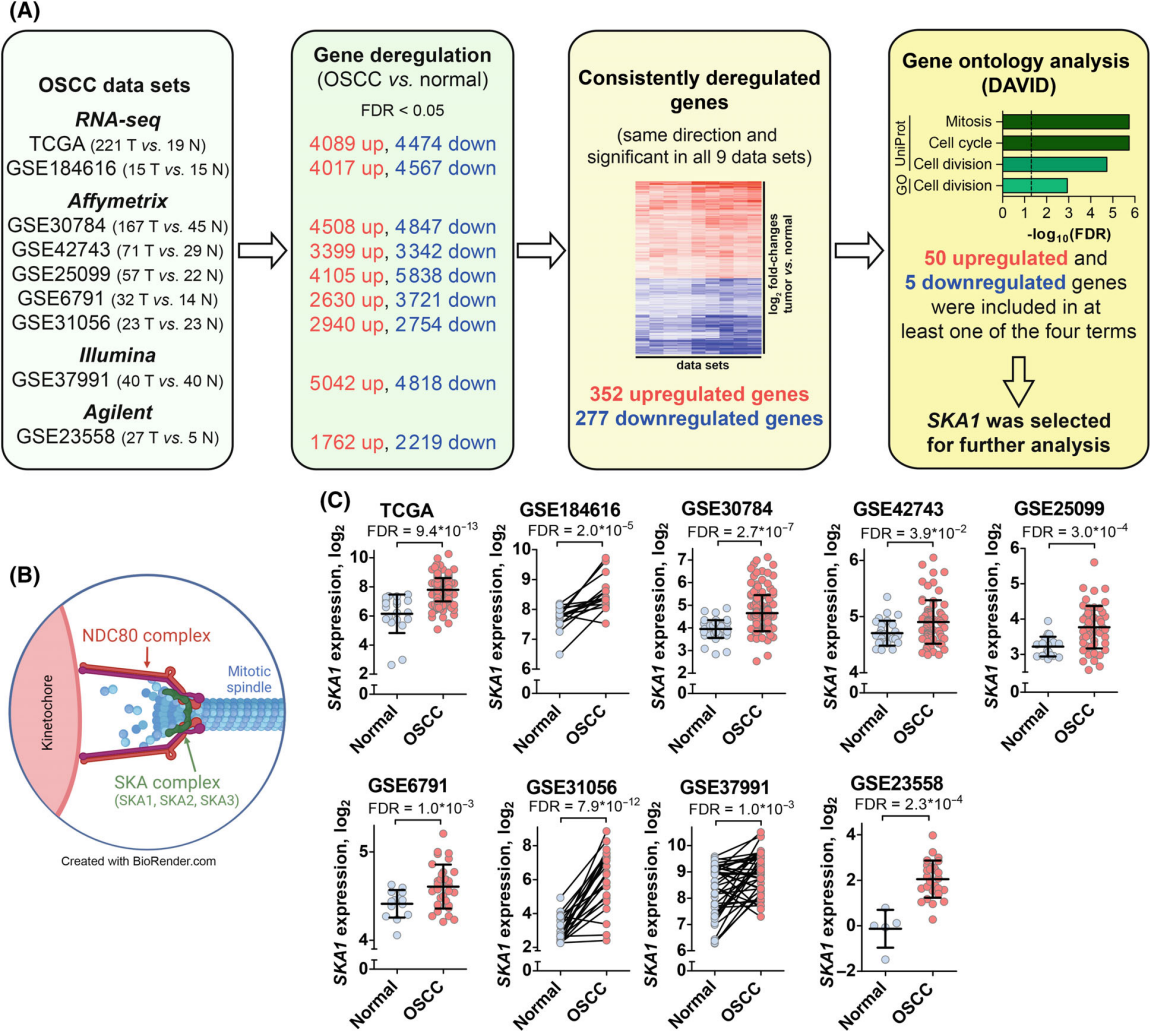
*SKA1* is consistently upregulated in OSCC *vs*. normal oral mucosa. (A) Schematic of bioinformatics analyses. Genes significantly (false discovery rate (FDR) < 0.05) and consistently dysregulated between oral squamous cell carcinoma (OSCC; T) and normal oral mucosa (N) samples were identified using nine publicly available gene expression datasets. Where applicable (TCGA dataset, GSE30784, GSE42743, and GSE6791), samples from sites other than the oral cavity or with known HPV^+^ status were excluded from the analyses, yielding the numbers of samples indicated in the leftmost panel. The resulting list of 629 consistently dysregulated genes was subjected to gene ontology (GO) analysis using DAVID. (B) Schematic drawing of the SKA complex at the connection between kinetochore, NDC80 complex, and mitotic spindle. Created in BioRender.com. Grandits, A. (2024) BioRender.com/k74k367. (C) *SKA1* mRNA levels in OSCC and normal mucosa samples in the nine datasets. Spaghetti plots and dot plots are used for datasets containing paired and unpaired samples, respectively. Means ± SD.

To identify key genes and processes relevant to OSCC pathogenesis, we used an unbiased bioinformatics approach and re‐analyzed nine publicly available genome‐wide gene expression datasets containing both OSCC and normal oral mucosa samples (Fig. 1A, Table S1). In total, 660 HPV‐negative OSCC and 211 normal oral mucosa samples that had been analyzed using different techniques (microarray, RNA‐seq) and platforms (Affymetrix, Illumina, Agilent) were included in the analyses. Genes that were significantly differentially expressed between OSCC and normal oral mucosa at an FDR < 0.05 were identified in all datasets individually. Intersection of the resulting gene lists yielded 352 and 277 unique genes that were consistently up‐ and downregulated, respectively, in all nine datasets (Fig. 1A, Table S3). GO analysis of these 629 consistently dysregulated genes revealed an annotation cluster comprising the UniProt keywords “mitosis”, “cell cycle”, and “cell division” and the GO term “cell division” as most highly and significantly enriched (Fig. 1A, Table S4). 55 of the 629 DEGs were associated with at least one of these four terms.

Based on a literature review of these 55 genes, *SKA1* was selected as an interesting candidate for further analysis because it had been characterized as an oncogene in other cancers [28, 30, 32, 34, 36, 37, 38, 39], but knowledge about its role in OSCC was scarce. SKA1 forms a heteromeric complex with SKA2 and SKA3 and plays a key role in the attachment of kinetochores to the mitotic spindle during metaphase [24, 25] (Fig. 1B). *SKA1* was overexpressed in various solid tumor entities [30, 31, 32, 33, 34], and a genome‐wide CRISPR screen (https://depmap.org/portal/) revealed that it had a strong impact on the proliferation of most cancer cell lines (median Chronos score = −0.67; Fig. S1. The Chronos algorithm accounts for various confounders in genome‐wide CRISPR screens; a score of −1 corresponds to the median of all common essential genes [53]). We therefore set out to study the function of *SKA1* in OSCC as well as in its premalignant precursor, OLP.

The log_2_ fold change (log_2_FC) of *SKA1* between OSCC and normal oral mucosa ranged from 0.2 to 3.1 in the different datasets (Fig. 1C). *SKA2* and *SKA3* were also significantly upregulated in OSCC *vs*. normal mucosa, but in only seven of the nine datasets and to a lesser extent than *SKA1* (log_2_FC 0.2–1.3 and 0.2–1.7 for *SKA2* and *SKA3*, respectively; Fig. S2A,B). Nevertheless, their Chronos scores suggest similarly important roles for proliferation of cancer cells (−0.75 and −0.69, respectively).

In summary, *SKA1*, and to a lesser extent its heteromeric partners *SKA2* and *SKA3*, are consistently upregulated in OSCC, and DepMap data point towards an oncogenic function of the SKA complex in this entity. Because it was most strongly and consistently dysregulated, we focused our further analyses on *SKA1*.

### 3.2 *SKA1* promotes proliferation and colony formation of OSCC cells

**Fig. 2:**
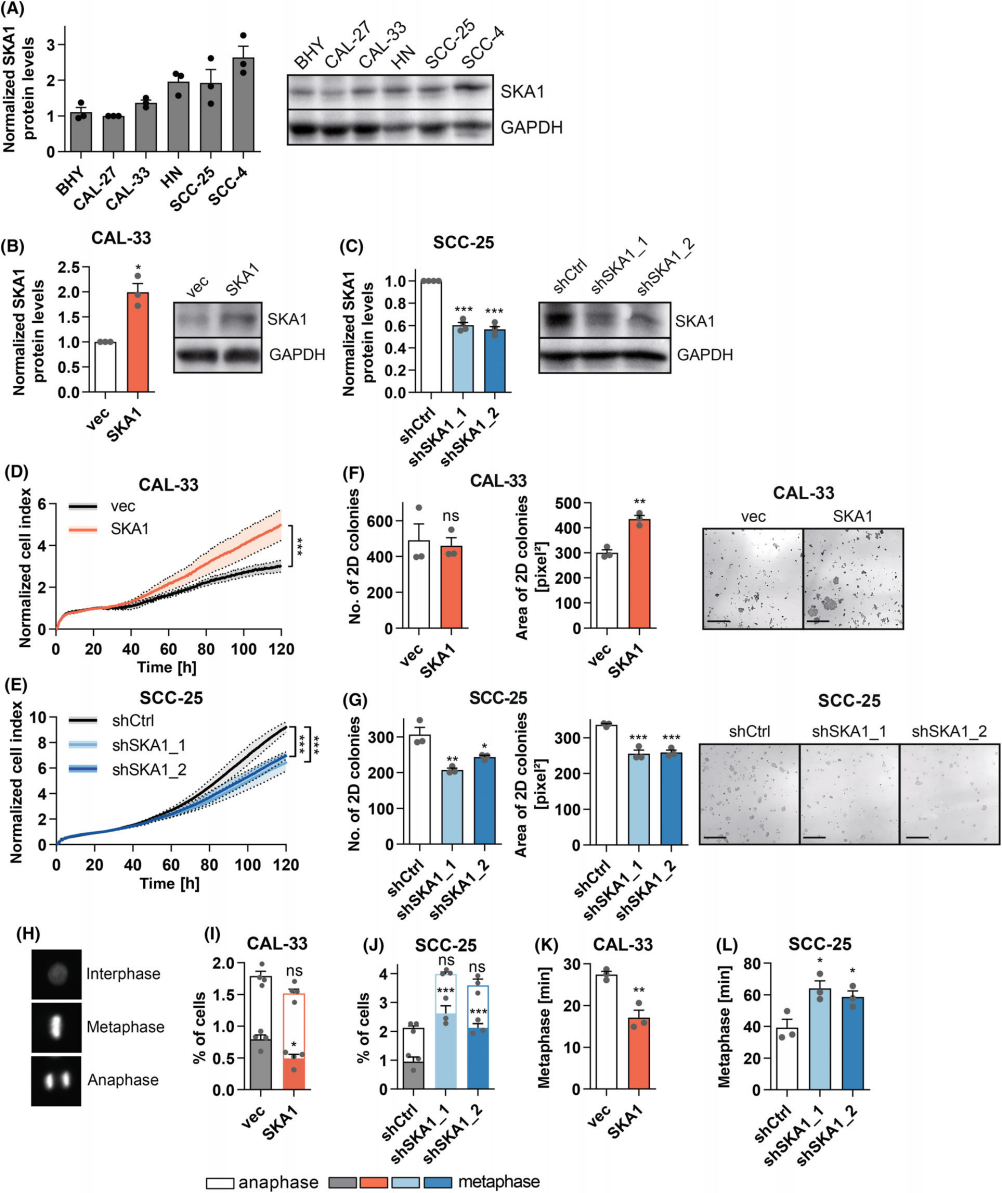
*SKA1* promotes proliferation and accelerates the progression through metaphase in OSCC cells. (A) SKA1 levels in the oral squamous cell carcinoma cell lines BHY, CAL‐27, CAL‐33, HN, SCC‐25, and SCC‐4 were determined by immunoblot analysis. (B) CAL‐33 cells were transduced with a retroviral vector containing the *SKA1* cDNA (SKA1) or with empty vector (vec). SKA1 overexpression was confirmed by immunoblot analysis. (C) SCC‐25 cells were transduced with lentiviral vectors containing *SKA1*‐specific shRNAs (shSKA1_1, shSKA1_2) or a control shRNA targeting *renilla luciferase* (shCtrl). Knockdown of SKA1 after shRNA induction by doxycycline was confirmed by immunoblot analysis. (A–C) Left panels, quantifications; right panels, representative experiments. Means + SEM, *n* = 3 (A, B) or 4 (C). *P < 0.05, ***P < 0.001; one‐sample *t*‐tests with Bonferroni correction. (D, E) Proliferation of CAL‐33_vec and CAL‐33_SKA1 cells (D), or of SCC‐25_shCtrl, SCC‐25_shSKA1_1, and SCC‐25_shSKA1_2 cells in the presence of doxycycline (E) was monitored using the xCELLigence system. Means ± SEM, *n* = 3. ***P < 0.001; *anova.lme* (r package). (F, G) 2D colony formation of CAL‐33_vec and CAL‐33_SKA1 cells (F), or of SCC‐25_shCtrl, SCC‐25_shSKA1_1, and SCC‐25_shSKA1_2 cells in the presence of doxycycline (G). Left: colony numbers, middle: colony areas, right: representative images. Means + SEM, *n* = 3. ns, not significant, *P < 0.05, **P < 0.01, ***P < 0.001; Student's *t*‐test (F), one‐way ANOVA followed by Dunnett's multiple comparison test (G). Scale bar = 3 mm. (H) Representative images of DAPI‐stained nuclei in different cell cycle phases. (I, J) Percentage of cells in metaphase and anaphase. CAL‐33_vec and CAL‐33_SKA1 cells (I), or doxycycline‐treated SCC‐25_shCtrl, SCC‐25_shSKA1_1, and SCC‐25_shSKA1_2 cells (J) were fixed and stained with DAPI. Nuclei were classified as shown in (H). Means + SEM, *n* = 3 (J) or 4 (I). ns, not significant, *P < 0.05, ***P < 0.001; two‐way ANOVA followed by Bonferroni's *post‐hoc* test. (K, L) Metaphase duration. CAL‐33_vec and CAL‐33_SKA1 cells (K), or doxycycline‐treated SCC‐25_shCtrl, SCC‐25_shSKA1_1, and SCC‐25_shSKA1_2 cells (L) were stained with Hoechst 33342 and metaphase duration was quantified using live‐cell imaging. Means + SEM, *n* = 3. *P < 0.05, **P < 0.01; Student's *t*‐test (K), one‐way ANOVA followed by Dunnett's multiple comparison test (L).

To investigate the roles of *SKA1* in OSCC, its expression levels were quantified in a panel of six human OSCC cell lines by immunoblot analysis (Fig. 2A) and qRT‐PCR (Fig. S3A). Based on their *SKA1* expression levels, CAL‐33 cells were selected for overexpression, and SCC‐25 cells, which have more favorable growth characteristics than SCC‐4 cells, for knockdown experiments. Retroviral transduction of CAL‐33 cells with a *SKA1* overexpression vector or with an empty vector as control yielded CAL‐33_SKA1 and CAL‐33_vec cells, respectively. Infection of SCC‐25 cells with lentiviral vectors containing a control shRNA (shCtrl) or one of two different shRNAs targeting *SKA1* (shSKA1_1, shSKA1_2) under the control of a doxycycline‐regulated promoter resulted in cell lines SCC‐25_shCtrl, SCC‐25_shSKA1_1, and SCC‐25_shSKA1_2. *SKA1* overexpression and knockdown were confirmed by immunoblot analysis (Fig. 2B,C) and qRT‐PCR (Fig. S3B,C). Experimental manipulation of *SKA1* expression had no effect on the mRNA levels of *SKA2* and *SKA3* (Fig. S3D,E). To test whether *SKA1* affected proliferation, cell numbers were monitored in real‐time by the xCELLigence assay. Overexpression of *SKA1* promoted, and its knockdown reduced, proliferation of OSCC cells (Fig. 2D,E). Along similar lines, in a 2D colony formation assay CAL‐33_SKA1 cells gave rise to larger colonies than CAL‐33_vec cells, and knockdown of *SKA1* in SCC‐25 cells reduced both colony size and number (Fig. 2F,G). The effects of *SKA1* on cell proliferation were not due to altered basal levels of cell death (Fig. S3F,G). In addition, experimental manipulation of *SKA1* expression did not significantly affect the distribution of cells in the G_0_/G_1_, S, and G_2_/M phases of the cell cycle (Fig. S3H,I). However, overexpression of *SKA1* decreased, and its knockdown increased, the proportion of cells in metaphase (Fig. 2H–J). This could reflect an inhibitory effect of *SKA1* on entry into mitosis (which would be at odds with its above‐described pro‐proliferative effect), or a *SKA1*‐mediated acceleration of the progression through metaphase (in line with the role of SKA1 as a mediator of the spindle‐kinetochore interaction). To discriminate between these possibilities, we assessed metaphase duration through live‐cell imaging. These experiments revealed that cells with high *SKA1* expression (CAL‐33_SKA1, SCC‐25_shCtrl) required markedly less time from alignment of the chromosomes at the metaphase plate to the initiation of chromosome segregation than the corresponding cells with low *SKA1* expression (CAL‐33_vec, SCC‐25_shSKA1_1, SCC‐25_shSKA1_2) (Fig. 2K,L, Fig. S4A,B).

In summary, *SKA1* accelerated the progression of OSCC cells through mitotic metaphase and promoted their proliferation.

### 3.3 *SKA1* enhances metastasis‐related properties of OSCC cells

**Fig. 3:**
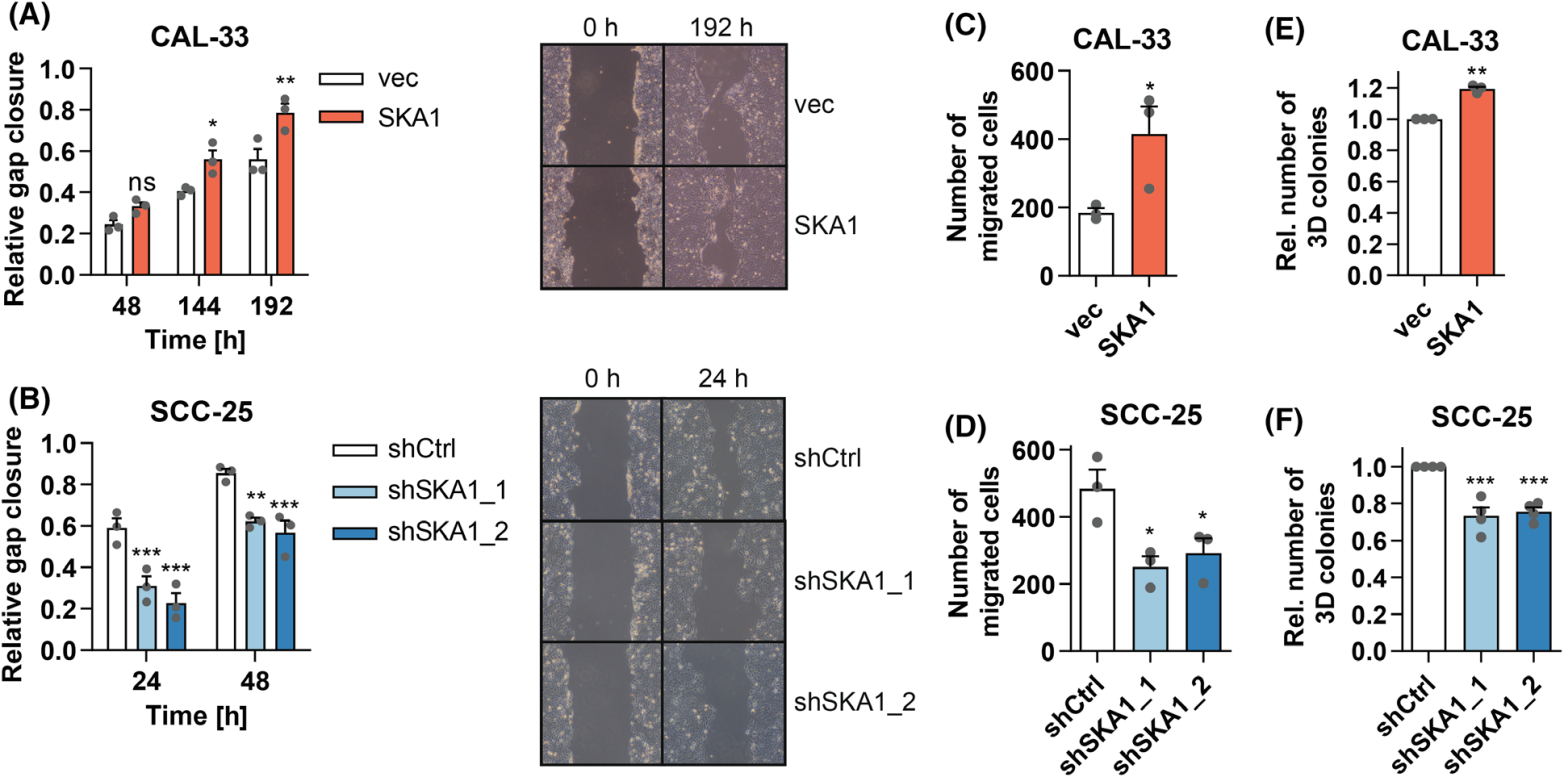
*SKA1* increases the migratory and 3D colony formation potential of OSCC cells. (A, B) Scratch assay. CAL‐33_vec and CAL‐33_SKA1 cells (A), or SCC‐25_shCtrl, SCC‐25_shSKA1_1, and SCC‐25_shSKA1_2 cells (B) were grown to 90% confluence in regular growth medium (+ doxycycline for SCC‐25 derivatives). The medium was changed to reduced serum conditions [0.2% or 10% fetal bovine serum (FBS) + doxycycline, respectively], scratches were introduced 24 h later, and gap closure was monitored at the indicated time points thereafter. Left panels: quantification; right panels: representative experiments (10x magnification). Means + SEM, *n* = 3. ns, not significant, *P < 0.05, **P < 0.01, ***P < 0.001; two‐way ANOVA followed by Bonferroni's *post‐hoc* test. (C, D) Transwell migration assay. CAL‐33_vec and CAL‐33_SKA1 cells (C), or SCC‐25_shCtrl, SCC‐25_shSKA1_1, and SCC‐25_shSKA1_2 cells (D) maintained under reduced serum conditions (+ doxycycline for SCC‐25 derivatives) for 1 day were seeded into Transwell® inserts and allowed to migrate towards medium with full serum supplementation (10% FBS or 20% FBS + doxycycline, respectively). After 24 h, cells were fixed and stained with trypan blue. Means + SEM, *n* = 3. *P < 0.05; Student's *t*‐test (C), one‐way ANOVA followed by Dunnett's multiple comparison test (D). (E, F) 3D colony formation on Matrigel®. CAL‐33_vec and CAL‐33_SKA1 cells (E), or doxycycline‐treated SCC‐25_shCtrl, SCC‐25_shSKA1_1, and SCC‐25_shSKA1_2 cells (F) were seeded onto Matrigel® (containing doxycycline in case of the SCC‐25 derivative lines) and colonies were imaged after 3 days. Means + SEM, *n* = 3 (E) or 4 (F). **P < 0.01, ***P < 0.001; one‐sample *t*‐tests with Bonferroni correction.

To query whether *SKA1* would also affect properties related to the formation of metastases, we assessed its effects on the abilities of OSCC cells to migrate and to regrow from single cells in semisolid media. In the wound healing assay, performed under conditions where cell proliferation as a potential confounder was minimal (Fig. S5A,B), overexpression of *SKA1* in CAL‐33 cells increased migration, and its knockdown in SCC‐25 cells reduced it (Fig. 3A,B). These results were confirmed by the Transwell® migration assay (Fig. 3C,D, Fig. S5C,D).

Moreover, CAL‐33_SKA1 cells gave rise to a larger number of 3D colonies on Matrigel® than CAL‐33_vec cells (Fig. 3E,F, Fig. S5E,F). Accordingly, SCC‐25_shSKA1_1 and SCC‐25_shSKA1_2 yielded lower numbers of colonies than SCC‐25_shCtrl.

Thus, *SKA1* promotes properties of OSCC cells that are associated with tumor progression and the formation of metastases.

### 3.4 *SKA1* expression increases radioresistance and mitigates radiation‐induced senescence, of OSCC cells

**Fig. 4:**
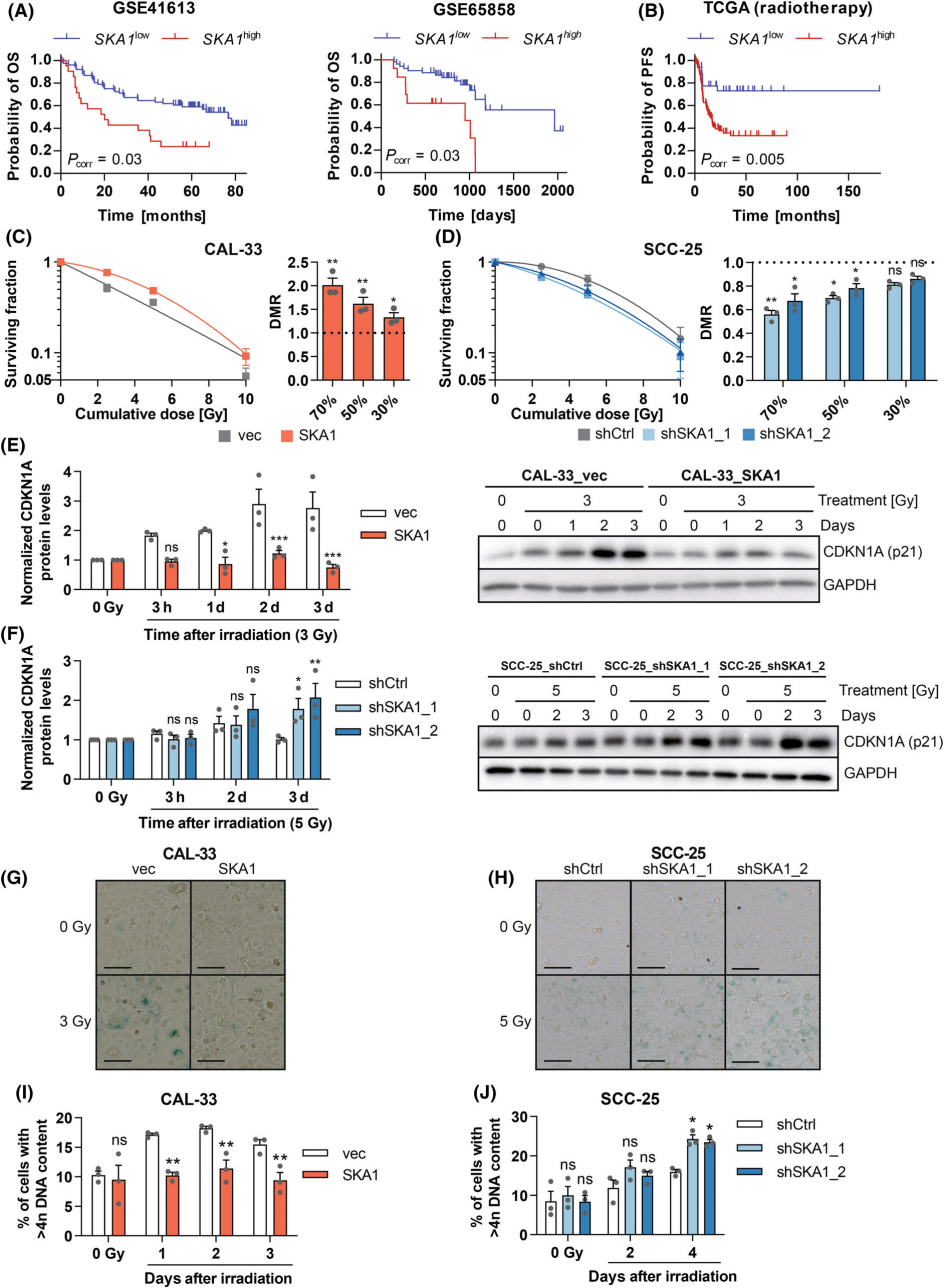
*SKA1* correlates with poor survival in OSCC and increases radioresistance of OSCC cell lines. (A, B) Kaplan–Meier curves relating the expression of *SKA1* to overall survival (OS) in oral squamous cell carcinoma (OSCC) datasets GSE41613 (*n* = 97) and GSE65858 (*n* = 65) (A), and to progression‐free survival (PFS) among radiation‐treated OSCC patients in the TCGA data (*n* = 126) (B). Optimal cutoffs for high *vs*. low *SKA1* expression were determined using maximally selected rank statistics (GSE41613: 5.44, GSE65858: 7.21, TCGA: 7.23). Significance was calculated using the log‐rank test; correction for determination of the optimal cutoff was performed according to Altman et al. [44]. (C, D) Radioresistance. CAL‐33_vec and CAL‐33_SKA1 cells (C), or doxycycline‐treated SCC‐25_shCtrl, SCC‐25_shSKA1_1, and SCC‐25_shSKA1_2 cells (D) were exposed to the indicated total radiation doses, delivered in equal fractions over 5 consecutive days. After 7 to 14 days, colonies were stained with trypan blue. Left panels, dose–response curves, right panels, dose‐modifying ratios (DMR). Means ± SEM, *n* = 3. ns, not significant, *P < 0.05, **P < 0.01; ratio *t*‐test. (E‐H) *SKA1* mitigates radiation‐induced senescence. CAL‐33_vec and CAL‐33_SKA1 (E, G), or doxycycline‐treated SCC‐25_shCtrl, SCC‐25_shSKA1_1, and SCC‐25_shSKA1_2 cells (F, H) were exposed to the indicated radiation dose and subjected to immunoblot analysis for the senescence marker CDKN1A (E, F), or stained for senescence‐associated β‐galactosidase (G, H). (E, F) Left panels, quantification, right panels, representative experiments. Means + SEM, *n* = 3. *P < 0.05, **P < 0.01, ***P < 0.001; two‐way ANOVA followed by Bonferroni's *post‐hoc* test. (G, H) Scale bar = 100 μm. (I, J) *SKA1* mitigates radiation‐induced hyperdiploidy. CAL‐33_vec and CAL‐33_SKA1 (I), or doxycycline‐treated SCC‐25_shCtrl, SCC‐25_shSKA1_1, and SCC‐25_shSKA1_2 cells (J) were exposed to a radiation dose of 3 or 5 Gy, respectively, and fixed and stained with DAPI on the indicated days thereafter. Cellular DNA content was assessed by flow cytometry. The proportion of cells with a > 4n DNA content is shown. Means + SEM, *n* = 3. ns, not significant, *P < 0.05, **P < 0.01; two‐way ANOVA followed by Bonferroni's *post‐hoc* test.

Next, we explored the relation between *SKA1* overexpression and various clinical parameters in OSCC. Corresponding information is provided in gene expression datasets GSE41613, GSE65858, and TCGA. *SKA1* expression was not significantly associated with sex, age, UICC stage, or smoking behavior (Table S5A–C), but high *SKA1* expression (defined by a threshold determined through maximally selected rank statistics) was associated with significantly shorter overall survival of OSCC patients in GSE41613 and GSE65858 (Fig. 4A). Univariable Cox regression analyses confirmed this finding, and multivariable analyses including *SKA1* expression and stage corroborated *SKA1* expression as an independent prognostic parameter (Tables S5A,B). Similarly, *SKA1* expression was significantly associated with reduced progression‐free survival in the TCGA dataset, but interestingly, only among patients that had received radiotherapy (Fig. 4B, Table S5C).

Because the association between *SKA1* expression and survival may at least in part reflect an impact of this gene on therapy responsiveness, we investigated whether *SKA1* affected the sensitivity of OSCC cell lines to the key therapeutic modalities, chemotherapy and radiation. Experimental manipulation of *SKA1* did not alter cellular responsiveness to cisplatin, paclitaxel, or docetaxel (data not shown). By contrast, overexpression of *SKA1* promoted, and its knockdown reduced, resistance to both single‐dose and fractionated radiation, as assessed by a 2D colony formation assay (Fig. S6A,B, Fig. 4C,D). This effect seemed to decrease at high radiation doses, most likely because the generally small numbers of colonies formed under these conditions obscured any differences.

*SKA1*‐mediated radioprotection could be related to a variety of biological processes including apoptosis, autophagy, DNA repair, and senescence [54, 55]. The levels of cleaved caspase 3 and of the autophagy marker LC3A/B were not affected by radiation and/or *SKA1* (Fig. S6C–F). *SKA1* expression levels also did not affect the initial number or the repair kinetics of DNA double strand breaks after irradiation as assessed via immunofluorescence for γH2AX (Fig. S6G,H). However, overexpression of *SKA1* inhibited, and its knockdown promoted, increases in cell size and in the expression of the senescence markers, CDKN1A and senescence‐associated β‐galactosidase, in response to a single radiation dose (Fig. 4E–H, Fig. S6I,J). Also, radiation‐induced increases in the proportion of hyperdiploid cells (*i*.*e*., cells with a > 4n DNA content) were mitigated by overexpression, and enhanced by knockdown, of *SKA1* (Fig. 4I,J).

In summary, overexpression of *SKA1* in OSCC was associated with shorter patient survival, which may be related to its abilities to promote radioresistance and mitigate radiation‐induced senescence in OSCC cells.

### 3.5 *SKA1* promotes tumor‐related properties in premalignant oral epithelial cells

**Fig. 5:**
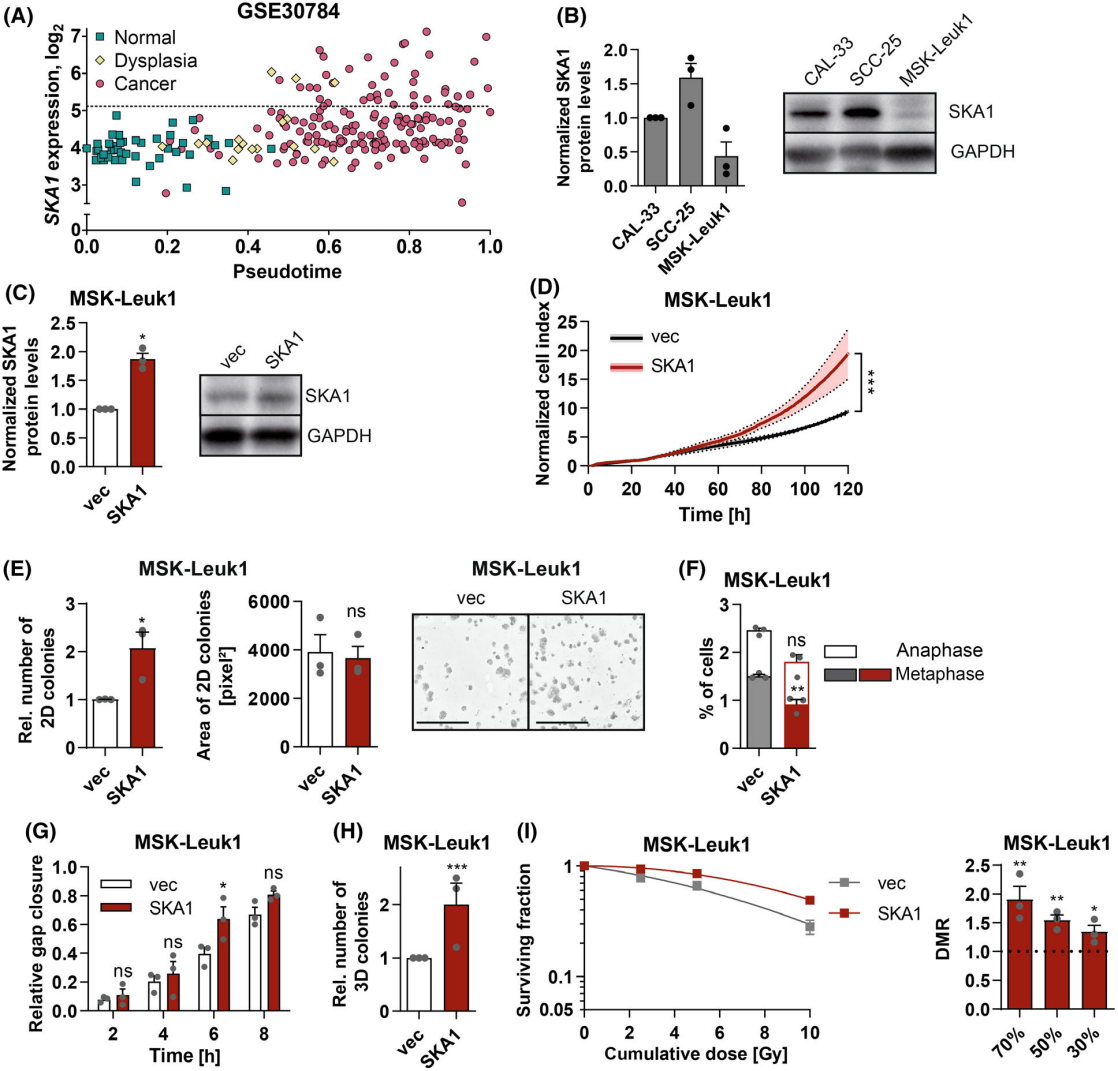
*SKA1 is* overexpressed and promotes tumor‐related properties, in oral dysplasia. (A) Relation between *SKA1* expression and pseudotime in normal oral mucosa (*n* = 45), oral dysplasia (*n* = 17), and oral squamous cell carcinoma (*n* = 167) (GSE30784). Dotted line, cutoff for *SKA1* overexpression. (B) SKA1 levels in CAL‐33, SCC‐25, and MSK‐Leuk1 cells were determined by immunoblot analysis. (C) Overexpression of SKA1 in MSK‐Leuk1_vec (vec) *vs*. MSK‐Leuk1_SKA1 (SKA1) cells was confirmed by immunoblot analysis. (B, C) Left panels, quantifications, right panels, representative experiments. Means + SEM, *n* = 3. *P < 0.05; one‐sample *t*‐test. (D) Proliferation of MSK‐Leuk1_vec and MSK‐Leuk1_SKA1 cells was monitored using the xCELLigence system. Data were normalized to the 24 h time point. Means ± SEM, *n* = 3. ***P < 0.001; *anova.lme* (r package). (E) 2D colony formation by MSK‐Leuk1_vec and MSK‐Leuk1_SKA1 cells. Left: relative colony numbers, middle: colony areas, right: representative experiment. Means + SEM, *n* = 3. ns, not significant, *P < 0.05; one‐sample *t*‐test. Scale bar = 8 mm (F) Percentage of fixed, DAPI‐stained MSK‐Leuk1_vec and MSK‐Leuk1_SKA1 cells in metaphase and anaphase. Nuclei were classified as shown in Fig. 2H. Means + SEM, *n* = 3. ns, not significant, **P < 0.01; two‐way ANOVA followed by Bonferroni's *post‐hoc* test. (G) Scratch assay. Scratches were introduced into confluent MSK‐Leuk1_vec and MSK‐Leuk1_SKA1 cell monolayers, and gap closure was monitored. Means + SEM, *n* = 3. ns, not significant, *P < 0.05; two‐way ANOVA followed by Bonferroni's *post‐hoc* test. (H) 3D colony formation in Matrigel®. Means + SEM, *n* = 3. ***P < 0.001; one‐sample *t*‐test. (I) Radioresistance. Sparsely seeded MSK‐Leuk1_vec and MSK‐Leuk1_SKA1 cells were exposed to the indicated total radiation doses, delivered in equal fractions over 5 consecutive days. After 7 days, colonies were stained with trypan blue. Left panels, dose–response curves, right panels, dose‐modifying ratios (DMR). Mean ± SEM, *n* = 3. *P < 0.05, **P < 0.01; ratio *t*‐test.

To investigate whether upregulation of *SKA1* was an early or a late event during the transformation from normal oral mucosa to OSCC, pseudotime analysis, which orders samples on a trajectory based on global gene expression patterns and facilitates a more refined estimation of the extent of malignant progression than simple grouping into normal, premalignant, and cancerous stages, was used [56]. Dataset GSE30784 contains 45 normal, 17 dysplastic, and 167 OSCC samples. Pseudotime analysis based on the 8000 most variably expressed genes arranged the samples in the expected order (normal—dysplastic—malignant), but also revealed substantial overlaps between these groups, consistent with a continuous progression from normal mucosa through dysplasia to malignancy (Fig. 5A). *SKA1* upregulation (defined as an expression higher than the mean + 3 standard deviations of normal samples) was observed in 18% and 23% of the dysplasia and OSCC samples, respectively. Among the dysplasia samples, it was observed exclusively in samples with advanced pseudotime scores similar to “early” OSCC samples, while among OSCC samples, it was already present from relatively early pseudotime scores onward (Fig. 5A). Therefore, we hypothesized that upregulation of *SKA1* may contribute to the transition from oral dysplasia to outright malignancy in a subset of patients.

To address this question experimentally, we used MSK‐Leuk1, a cell line established from an oral dysplasia [45]. Immunoblot analysis revealed that the expression of SKA1 in MSK‐Leuk1 cells was even lower than in CAL‐33 cells (Fig. 5B; corresponding qRT‐PCR analysis, Fig. S7A); hence, we overexpressed *SKA1* in these cells (Fig. 5C, Fig. S7B). Other than with the OSCC cell lines, *SKA1* overexpression in MSK‐Leuk1 was accompanied by increased mRNA levels of *SKA2* and *SKA3* (Fig. S7C). Neither basal rates of cell death nor cell cycle distribution were affected by *SKA1* expression (Fig. S7D,E), yet MSK‐Leuk1_SKA1 cells proliferated faster, formed more 2D colonies, had a lower proportion of cells in metaphase, migrated faster, and gave rise to a higher number of colonies in Matrigel® than MSK‐Leuk1_vec cells (Fig. 5D–H, Fig. S7F,G). Moreover, *SKA1* augmented radioresistance of MSK‐Leuk1 cells (Fig. 5I, Fig. S7H).

In summary, *SKA1* was upregulated in a subset of patients already at the transition from premalignancy to outright OSCC, and experimental expression of *SKA1* augmented tumor‐relevant properties, including radioresistance, of premalignant MSK‐Leuk1 cells. Thus, *SKA1* upregulation may make a relevant contribution to the transformation from oral dysplasia to outright malignancy.

### 3.6 *SKA1* activates distinct gene expression patterns in OSCC and oral dysplastic cells

**Fig. 6:**
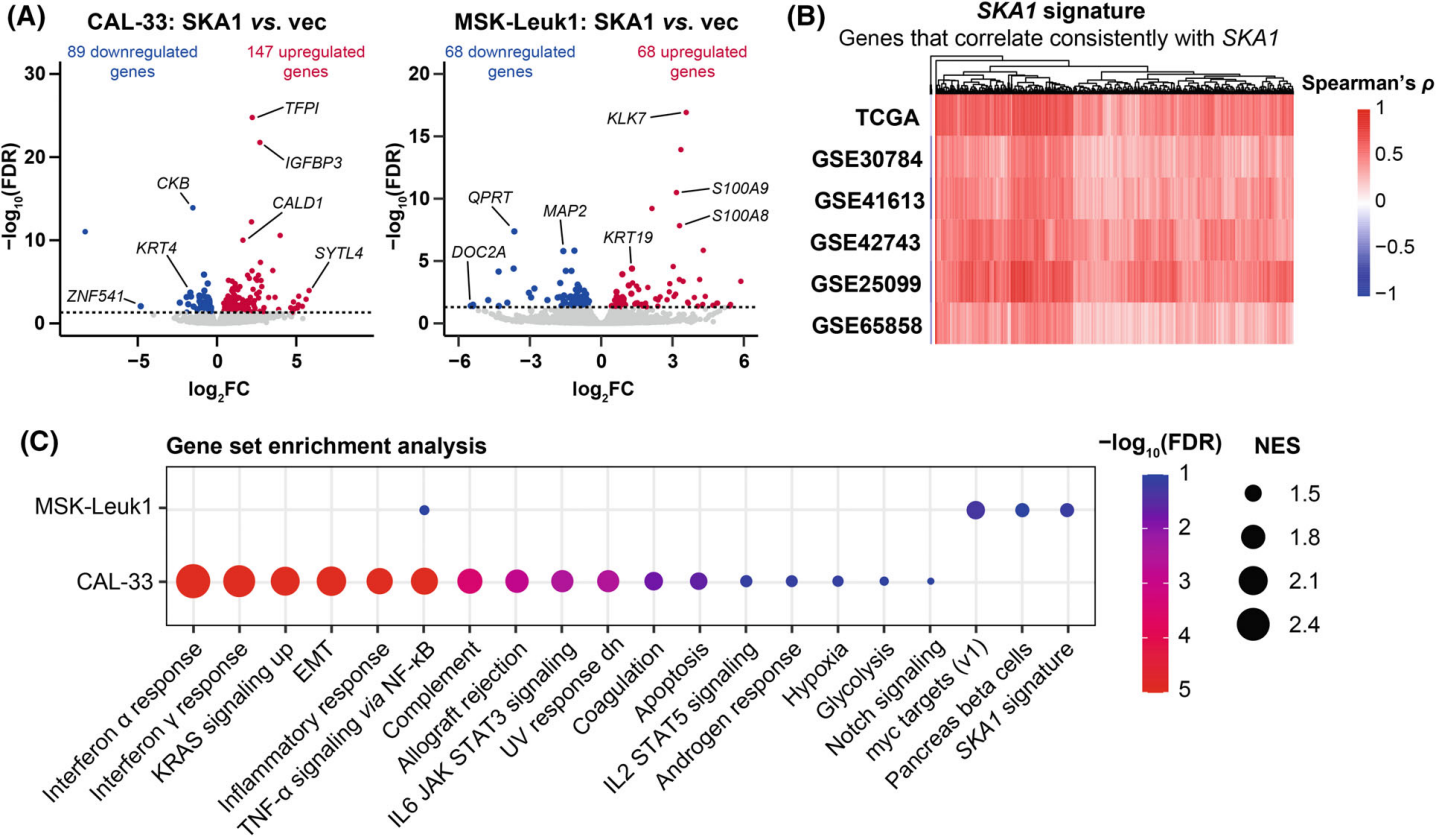
*SKA1* activates expression of distinct gene signatures in OSCC and premalignant cells. (A) Volcano plots illustrating genes differentially expressed between CAL‐33_vec (*n* = 4) and CAL‐33_SKA1 (*n* = 4), and between MSK‐Leuk1_vec (*n* = 3) and MSK‐Leuk1_SKA1 (*n* = 4). Gene expression was determined by RNA‐seq. Genes upregulated and downregulated at a false discovery rate (FDR) < 0.05 are shown in red and blue, respectively. Dotted line, FDR = 0.05. (B) Heatmap showing Spearman's ρ correlation coefficients of the 549 genes that consistently correlated with *SKA1* expression (FDR < 0.05) in datasets containing more than 50 oral squamous cell carcinoma samples (“*SKA1* signature”). (C) Gene set enrichment analysis of genes differentially expressed between CAL‐33_vec and CAL‐33_SKA1, and between MSK‐Leuk1_vec and MSK‐Leuk1_SKA1. EMT, epithelial‐mesenchymal transition; NES, normalized enrichment score.

Finally, we investigated transcriptional changes in response to *SKA1* overexpression in OSCC cells and in premalignant cells using RNA‐seq. 236 genes were significantly differently expressed between CAL‐33_vec and CAL‐33_SKA1 cells (147 up‐ and 89 downregulated), and 136 between MSK‐Leuk1_vec and MSK‐Leuk1_SKA1 cells (68 up‐ and 68 downregulated) (Fig. 6A, Tables S6A,B). Unexpectedly, only seven genes responded to *SKA1* overexpression in both cell lines. To explore the differences between the two signatures on a functional level, GSEA was performed using predefined hallmark gene sets as well as a custom set of 549 genes that were significantly (FDR < 0.05) and consistently correlated with *SKA1* in six datasets containing at least 50 OSCC samples (termed “*SKA1* signature”; Fig. 6B, Table S7). The *SKA1* signature contained many cell division‐associated genes including *SKA2* and *SKA3*, the NDC80 complex components NDC80 kinetochore complex component (*NDC80*), NUF2 component of NDC80 kinetochore complex (*NUF2*), SPC24 component of NDC80 kinetochore complex (*SPC24*), and SPC25 component of NDC80 kinetochore complex (*SPC25*), and kinesin family member 23 (*KIF23*), a key contributor to cleavage furrow ingression. Interestingly, overexpression of *SKA1* activated different functional programs in OSCC and premalignant cells. In CAL‐33 cells, gene sets reflecting the activation of the immune response and EMT reached top scores (Fig. 6C, Table S8A). The *SKA1* signature was not significantly enriched (FDR > 0.1), possibly due to an already high expression of several of its constituent genes in the absence of *SKA1* overexpression (Fig. S7I). In MSK‐Leuk1 cells, on the other hand, the proliferation‐associated gene sets “myc targets” and the *SKA1* signature were among the most significantly enriched pathways (the comparably moderate enrichment was probably at least partially due to the relatively low number of DEGs in this cell line). All elements of the SKA and NDC80 complexes, except for *SPC24*, were within the leading‐edge subset of the *SKA1* signature (Table S8B). This is in line with our above‐described finding that the overexpression of *SKA1* led to upregulation of *SKA2* and *SKA3* in MSK‐Leuk1, but not CAL‐33 cells (Figs S3D,E and S7C). Similarly, upregulation of *NDC80* and *KIF23* in response to *SKA1* overexpression in MSK‐Leuk1 cells could be confirmed by qRT‐PCR (Fig. S7C).

In summary, overexpression of *SKA1* led to upregulation of functionally diverse gene signatures in OSCC (CAL‐33) and oral premalignant (MSK‐Leuk1) cells, potentially reflecting different roles in early and later stages of tumorigenesis.

## 4 Discussion

In this study, we describe the oncogenic functions of *SKA1*, a gene with key roles in cell cycle progression and capable of promoting tumor formation in mice, in OSCC, a frequent and aggressive tumor entity with limited therapeutic options [1, 2, 28, 36]. *SKA1* emerged as a compelling candidate gene from a comprehensive bioinformatics screen employing nine publicly available datasets for the identification of genes consistently dysregulated in OSCC as compared to normal oral mucosa. Notably, while similar methodologies have been previously reported [57, 58, 59], our study stands out due to its utilization of one of the largest numbers of datasets and the by far largest number of samples (660 OSCC samples *vs*. 341 in the second largest study [57]). The diversity of techniques (microarray, RNA‐seq) and platforms (Affymetrix, Agilent, Illumina) used to generate these datasets safeguards against potential technical artifacts. Between 3981 and 9355 genes were significantly differentially expressed between tumor and control samples in the different datasets, and 629 of these (352 up‐ and 277 downregulated) were shared between all datasets (while less than one shared upregulated or downregulated gene would be expected by chance). The GO terms most significantly enriched among the 629 consistently dysregulated genes were related to cell division and mitosis. While this concurs with sustained proliferative signaling representing one of the hallmarks of cancer [60], other OSCC studies identified terms related to immune response and extracellular matrix, which ranked lower and very low, respectively, in our analysis, as most significantly enriched [57, 58, 59]. Notably, these studies defined DEGs based on fold‐change cutoffs in addition to statistical significance. In contrast, we used only the latter criterion because the biological consequences of gene expression changes are not necessarily related to their magnitude, so that the use of an arbitrary fold‐change cutoff may lead to a loss of information [52]. Indeed, a GO analysis on only those 110 of our 623 DEGs that passed a two‐fold change cutoff yielded terms related to extracellular space and immune responses as most significantly enriched, while the—indisputably cancer‐relevant—cell cycle‐related terms were lost (Table S9).

From the pool of mitosis‐related genes consistently upregulated in OSCC, we prioritized *SKA1* for functional analysis. Despite its established oncogenic functions in other tumor entities, its role in OSCC had been characterized only to a very limited extent. Zhang et al. [61] used a single shRNA to knock down *SKA1* in CAL‐27 cells, which, in our experiments, exhibited some of the lowest levels of endogenous SKA1 among OSCC cell lines. Nevertheless, consistent with our results they found that the knockdown of *SKA1* decreased proliferation and colony formation [61]. They also reported significant increases in the proportions of cells in the G_0_/G_1_ and G_2_/M phases of the cell cycle, as well as a minuscule, but significant increase in the proportion of apoptotic (sub‐G_1_) cells, findings which were not corroborated by our experiments. Our studies employed two independent shRNAs to rule out off‐target effects and included a *SKA1* overexpression model, which showed effects complementary to those of the knockdown model in all assays. We further extended the previous report by showing that *SKA1* reduced metaphase duration and promoted cell migration. In contrast to what has been reported for non‐small cell lung cancer and osteosarcoma [29, 39], *SKA1* did not affect responsiveness to chemotherapy (cisplatin, paclitaxel, docetaxel) in OSCC cell lines. However, *SKA1* overexpression increased, and its knockdown decreased, resistance to radiation, an effect that to the best of our knowledge has not been reported before. *SKA1*‐mediated radioresistance is likely to be at least in part due to a decrease in radiation‐induced senescence, assessed via increases in cell size and the expression of CDKN1A and SA‐βgal. Moreover, EMT is associated with resistance to therapy, including radiation [62], hence the impact of *SKA1* on radiation resistance may also be related to its ability to promote EMT as reflected by enhanced migratory capacity and the enrichment of EMT‐related gene expression patterns. Unresectable OSCC patients are usually treated with 70 Gy applied in a fractionation regimen over a period of several weeks [2]. In our cell line models, we partially mimicked this setting by applying cumulative doses of up to 10 Gy in five equal fractions on consecutive days, corresponding to 1 week of the 7‐week radiotherapy regimen for OSCC. Over the course of a typical therapeutic regimen, the *SKA1*‐mediated radioresistance observed in these experiments may well accumulate to clinically relevant levels. Supporting this notion, in the TCGA dataset, which to our knowledge is the only publicly available OSCC dataset providing both survival data and detailed therapy information, high *SKA1* expression was negatively associated with progression‐free survival in patients that had received radiotherapy either alone or as part of a combination therapy.

Because experimental expression of *SKA1* had been reported to promote malignant transformation of immortalized, but non‐tumorigenic prostate epithelial cells [28], we queried its expression and function also in oral premalignancies. While immunohistochemistry had shown SKA1 to be expressed at significantly higher levels in six prostate intraepithelial neoplasia samples compared to normal controls [28], pseudotime analysis on a substantially larger number of samples from the public domain and using the continuous parameter mRNA expression revealed a more refined picture in oral premalignancies: *SKA1* expression varied between samples and was high only among those whose pseudotime score corresponded to that of outright OSCC samples. On the other hand, among OSCC samples, *SKA1* was overexpressed already in some of those with relatively low pseudotime scores. Functional assays confirmed that *SKA1* promoted tumor‐relevant properties, including radioresistance, in OLP (MSK‐Leuk1) cells in a similar manner as in OSCC (CAL‐33) cells. Nevertheless, its experimental overexpression provoked different gene expression changes in these two cell line models: in the premalignant cell line, genes related to proliferation—an early requirement during tumorigenesis—were enriched among *SKA1*‐induced DEGs, while in the OSCC cell line, terms related to more advanced oncogenic properties, like EMT and immune responses, were enriched.

## 5 Conclusions

In this study, we show that *SKA1* functions as an oncogene and augments cell proliferation and migration in OSCC. Unlike its role in other tumor types, it does not appear to promote chemotherapy resistance, but it does enhance radioresistance, a function that, to the best of our knowledge, has never been reported before, and that may be related to its ability to reduce radiation‐induced senescence. *SKA1* expression could thus serve as a valuable marker of radioresistance in OSCC. Moreover, considering the success of targeting cell cycle proteins in cancer therapy, exemplified by the development of CDK4/6 inhibitors [63], SKA1 presents itself as an attractive therapeutic target. While it may not be an obvious candidate compared to protein kinases, synthetic dosage lethality [64] and/or proteolysis‐targeting chimeras (PROTACs) [65] could offer avenues for tackling this oncoprotein in OSCC and other cancers.

## Conflict of interest

ASB has research support from Daiichi Sankyo, Roche and honoraria for lectures, consultation, or advisory board participation from Roche, Bristol‐Meyers Squibb, Merck KGaA, Daiichi Sankyo, AstraZeneca, CeCaVa, Seagen, Alexion, Servier as well as travel support from Roche, Amgen and AbbVie. FE has received travel grants and honoraria for presentations by Dr. Sennewald Medizintechnik and has participated in advisory boards for Servier. TF has received honoraria from and/or was advisor for MSD, Merck KGaA, Bristol‐Meyers Squibb, Boehringer Ingelheim, Roche, Sanofi, Amgen, Takeda, Invios, Janssen, and Ely Lilly. All other authors declare that they do not have any conflict of interest.

## Author contributions

Conceptualization, AMG, RW; methodology, AMG, BAR, RWa, PK, FE, RW; validation, AMG, RW; formal analysis, AMG; investigation, AMG, BAR, RWa, RW; writing‐original draft preparation, AMG, RW; writing‐review and editing: all authors; visualization, AMG; supervision, ASB, TF and RW; project administration, TF and RW; funding acquisition, TF. All authors have read and agreed to the published version of the manuscript.

### Peer review

The peer review history for this article is available at https://www.webofscience.com/api/gateway/wos/peer‐review/10.1002/1878‐0261.13780.

## Supporting information


**Fig. S1.** Loss of *SKA1* impedes the proliferation of cancer cell lines.
**Fig. S2.** *SKA2* and *SKA3* are upregulated in OSCC *vs*. normal oral mucosa, but to a lesser extent than *SKA1*.
**Fig. S3.** *SKA1* expression does not alter the rate of spontaneous cell death or the cell cycle distribution of OSCC cell lines.
**Fig. S4.** *SKA1* promotes progression of OSCC cell lines through metaphase.
**Fig. S5.** *SKA1* promotes migration and 3D colony formation of OSCC cell lines.
**Fig. S6.** *SKA1* does not affect apoptosis, autophagy, or the formation and repair of DNA double strand breaks in response to irradiation, but reduces radiation‐induced senescence.
**Fig. S7.** *SKA1* promotes malignant properties in an OLP cell line.

**Table S1.** Overview of publicly available datasets used for identification of genes consistently dysregulated in OSCC, for survival analysis, and for pseudotime analysis.
**Table S2.** Oligonucleotides and primers used for cloning of shRNAs and of the *SKA1* cDNA, and qRT‐PCR primers.
**Table S3.** List of 629 genes that were consistently dysregulated between OSCC and normal oral mucosa in all 9 datasets.
**Table S4.** Results of the gene ontology analysis of the 629 genes consistently dysregulated between OSCC and normal oral mucosa (Table S3).
**Table S5A.** Correlation between *SKA1* expression and clinical parameters, and Cox regression analysis in OSCC dataset GSE41613.
**Table S5B.** Correlation between *SKA1* expression and clinical parameters, and Cox regression analysis of the 65 OSCC patients contained in dataset GSE65858.
**Table S5C.** Correlation between *SKA1* expression and clinical parameters, and Cox regression analysis for the 126 patients in the TCGA OSCC dataset who had received radiotherapy.
**Table S6A.** Genes differentially expressed between CAL‐33_vec and CAL‐33_SKA1 cells (RNA‐seq analysis).
**Table S6B.** Genes differentially expressed between MSK‐Leuk1_vec and MSK‐Leuk1_SKA1 cells (RNA‐seq analysis).
**Table S7.** List of the 549 genes that were consistently correlated with *SKA1* in the datasets TCGA, GSE30784, GSE41613, GSE42743, GSE25099, and GSE65858.
**Table S8A.** Results of the gene set enrichment analysis of the DEGs between CAL‐33_vec and CAL‐33_SKA1 cells (Supplementary Table S6A).
**Table S8B.** Results of the gene set enrichment analysis of the DEGs between MSK‐Leuk1_vec and MSK‐Leuk1_SKA1 cells (Supplementary Table S6B).
**Table S9.** Results of the gene ontology analysis of the 110 genes that are consistently dysregulated between OSCC and normal oral mucosa with a log_2_ fold change of >1.

## Data Availability

RNA‐seq data were deposited in the Gene Expression Omnibus (accession number GSE262993). Materials to which the authors' institution holds legal rights are available upon request.
