# Supplementary material for: SKA1 promotes oncogenic properties in oral dysplasia and oral squamous cell carcinoma, and augments resistance to radiotherapy
Source: Mol Oncol. 2024 Dec 10;19(4):1054–74. doi: 10.1002/1878-0261.13780 (PMC11977640; doi:10.1002/1878-0261.13780)
Supplement: Supplementary file 1 — Fig. S1. Loss of SKA1 impedes the proliferation of cancer cell lines. Fig. S2. SKA2 and SKA3 are upregulated in OSCC vs. normal oral mucosa, but to a lesser extent than SKA1. Fig. S3. SKA1 expression does not alter the rate of spontaneous cell death or the cell cycle distribution of OSCC cell lines. Fig. S4. SKA1 promotes progression of OSCC cell lines through metaphase. Fig. S5. SKA1 promotes migration and 3D colony formation of OSCC cell lines. Fig. S6. SKA1 does not affect apoptosis, autophagy, or the formation and repair of DNA double strand breaks in response to irradiation, but reduces radiation‐induced senescence. Fig. S7. SKA1 promotes malignant properties in an OLP cell line. [file MOL2-19-1054-s002.docx]

Supplementary information for manuscript:

***SKA1* promotes oncogenic properties in oral dysplasia and oral squamous cell carcinoma and augments resistance to radiotherapy**

**This supplementary information includes**

[Supplementary Figure Legends 2](#_Toc176882893)

[Supplementary Figures S1 to S7 6](#_Toc176882894)

**Supplementary Figure Legends**

**Supplementary Figure S1: Loss of *SKA1* impedes the proliferation of cancer cell lines.** Chronos scores for *SKA1* in 380 human epithelial cancer cell lines from the DepMap CRISPR knockout database (23Q2, <https://depmap.org/portal/>). A negative Chronos score indicates cell depletion upon CRISPR-mediated gene knockout; –1 corresponds to the median of all common essential genes.

**Supplementary Figure S2: *SKA2* and *SKA3* are upregulated in OSCC *vs*. normal oral mucosa, but to a lesser extent than *SKA1*.** (A,B) *SKA2* (A) and *SKA3* (B) mRNA levels in OSCC and normal oral mucosa samples in the nine data sets used for candidate gene identification. Spaghetti plots and dot plots are used for data sets containing paired and unpaired samples, respectively. FDRs < 0.05 are highlighted in green, bold letters.

**Supplementary Figure S3: *SKA1* expression does not alter the rate of spontaneous cell death or the cell cycle distribution of OSCC cell lines.** (A) *SKA1* mRNA levels in six OSCC cell lines were determined by qRT‑PCR and normalized to those of *β-2-microglobulin* and CAL‑27 using the ΔΔC_T_ method. Means + SEM, n = 3. (B) CAL‑33 cells were transduced with a retroviral vector containing the *SKA1* cDNA (SKA1) or with empty vector (vec). *SKA1* overexpression was confirmed by qRT‑PCR. Means + SEM, n = 3. ** *p* < 0.01. (C) SCC‑25 cells were transduced with lentiviral vectors containing *SKA1*-specific shRNAs (shSKA1_1, shSKA1_2) or a control shRNA targeting *renilla luciferase* (shCtrl). Knockdown of *SKA1* after shRNA induction by doxycycline (doxy) was confirmed by qRT‑PCR. Means + SEM, n = 3. ns, not significant, * *p* < 0.05. (D,E) *SKA2* (D) and *SKA3* (E) mRNA levels were determined by qRT‑PCR in CAL‑33_vec and CAL‑33_SKA1 cells (left panels), as well as in SCC-25_shCtrl, SCC‑25_shSKA1_1, and SCC‑25_shSKA1_2 cells (right panels), and normalized to those of *β-2-microglobulin* and of the respective control cell lines using the ΔΔC_T_ method. Means + SEM, n = 3. ns, not significant. (F,G) Cell death assay. CAL‑33_vec and CAL‑33_SKA1 cells (F), or SCC-25_shCtrl, SCC‑25_shSKA1_1, and SCC‑25_shSKA1_2 cells (G) were stained with Annexin V and DAPI and analyzed by flow cytometry. Means + SEM, n = 3. ns, not significant. (H,I) Cell cycle analysis. CAL‑33_vec and CAL‑33_SKA1 cells (H), or SCC-25_shCtrl, SCC‑25_shSKA1_1, and SCC‑25_shSKA1_2 cells (I) were fixed, stained with DAPI, and subjected to flow cytometry. Data were analyzed using FlowJo v10.0.7r2. Means + SEM, n = 3. ns, not significant.

**Supplementary Figure S4: *SKA1* promotes progression of OSCC cell lines through metaphase.** (A,B) CAL‑33_vec and CAL‑33_SKA1 cells (A), or SCC‑25_shCtrl, SCC‑25_shSKA1_1, and SCC‑25_shSKA1_2 cells (B) were stained with Hoechst 33342 and the time from alignment of the chromosomes at the metaphase plate to the initiation of chromosome segregation was determined using live cell imaging. Representative experiments (corresponds to **Fig. 2K,L**).

**Supplementary Figure S5: *SKA1* promotes migration and 3D colony formation of OSCC cell lines.** (A,B) Proliferation of CAL‑33_vec and CAL‑33_SKA1 cells (A), or SCC‑25_shCtrl, SCC‑25_shSKA1_1, and SCC‑25_shSKA1_2 cells (B) under reduced serum conditions (0.2 and 10% FBS, respectively) as used for the scratch assay. Metabolic activity was used as a surrogate for cell number. Means + SEM, n = 3. ns, not significant. (C,D) Transwell migration assay. CAL‑33_vec and CAL‑33_SKA1 cells (C), or SCC‑25_shCtrl, SCC‑25_shSKA1_1, and SCC‑25_shSKA1_2 cells (D) maintained under reduced serum conditions for 1 day were seeded into Transwell® inserts and allowed to migrate towards medium with full serum supplementation (10% or 20% FBS, respectively). After 24 h, cells were fixed and stained with trypan blue. Representative experiments (corresponds to **Figure 3C,D**). (E,F) 3D colony formation on Matrigel®. CAL‑33_vec and CAL‑33_SKA1 cells (E), or SCC‑25_shCtrl, SCC‑25_shSKA1_1, and SCC‑25_shSKA1_2 cells (F) were seeded on a layer of Matrigel® and colonies were imaged after 3 days. Scale bar = 1 mm. Representative experiments (corresponds to **Figure 3E,F**).

**Supplementary Figure S6: *SKA1* does not affect apoptosis, autophagy, or the formation and repair of DNA double strand breaks in response to irradiation, but reduces radiation-induced senescence.** (A-B) Radioresistance. CAL‑33_vec and CAL‑33_SKA1 cells (A), or doxycycline-treated SCC-25_shCtrl, SCC‑25_shSKA1_1, and SCC‑25_shSKA1_2 cells (B) were exposed to the indicated (single) radiation doses. After 7 to 14 days, colonies were stained with trypan blue. Left panels, dose-response curves, right panels, dose-modifying ratios (DMR). Means ± SEM, n = 3. ns, not significant, * *p* < 0.05, ** *p* <0.01 (ratio *t*‑test). (C‑J) CAL‑33_vec and CAL‑33_SKA1 cells (C,E,G,I), or doxycycline-treated SCC‑25_shCtrl, SCC‑25_shSKA1_1, and SCC‑25_shSKA1_2 cells (D,F,H,J) were exposed to the indicated radiation doses. (C,D) Immunoblot analysis for cleaved caspase-3. (E,F) Immunoblot analysis for the autophagy marker LC3A/B. (G,H) Immunofluorescence analysis of γH2AX foci at the indicated times after irradiation. Left panels, quantifications; right panels, representative images. Means + SEM, n = 3. ns, not significant. Scale bar = 2.5 µm. (I,J) Radiation-induced increase in cell size. Irradiated cells were incubated for two days, stained with phalloidin (green) and DAPI (blue), and imaged to determine cell size. Left panels, quantifications; right panels, representative images. Scale bar = 150 µm. Means + SEM, n = 3. ns, not significant; *, *p* < 0.05; **, *p* < 0.01.

**Supplementary Figure S7: *SKA1* promotes malignant properties in an OLP cell line.** (A) *SKA1* mRNA levels in CAL-33, SCC-25, and MSK-Leuk1 cells were determined by qRT‑PCR and normalized to those of *β-2-microglobulin* and CAL-33 using the ΔΔC_T_ method. Means + SEM, n = 3. (B) *SKA1* overexpression in *SKA1*- *vs*. empty vector-transduced MSK-Leuk1 cells was confirmed by qRT‑PCR. Means + SEM, n = 3. ** *p* < 0.01. (C) *SKA2*, *SKA3*, *NDC80*, and *KIF23* mRNA levels in MSK‑Leuk1_vec and MSK‑Leuk1_SKA1 cells were quantified by qRT‑PCR. Means + SEM, n = 3. * *p* < 0.05, ** *p* < 0.01. (D) Cell death assay. MSK‑Leuk1_vec and MSK‑Leuk1_SKA1 cells were stained with Annexin V and DAPI and analyzed by flow cytometry. Means + SEM, n = 3. ns, not significant. (E) Cell cycle analysis. MSK‑Leuk1_vec and MSK‑Leuk1_SKA1 cells were fixed, stained with DAPI, and subjected to flow cytometry. Data were analyzed using FlowJo v10.0.7r2. Means + SEM, n = 3. ns, not significant. (F) Scratch assay. MSK‑Leuk1_vec and MSK‑Leuk1_SKA1 cells were grown to confluence and scratches were introduced. Gap closure was monitored at several time points thereafter. Representative experiment (corresponds to **Figure 5G**). (G) 3D colony formation on Matrigel®. MSK‑Leuk1_vec and MSK‑Leuk1_SKA1 were seeded on top of a layer of Matrigel® and 3D colonies were imaged after 5 days. Scale bar = 1 mm. Representative experiment (corresponds to **Figure 5H**). (H) Radiation-induced increase in cell size. MSK‑Leuk1_vec and MSK‑Leuk1_SKA1 cells were irradiated with 0 and 3 Gy and incubated for 2 days. Cells were fixed, stained with phalloidin (green) and DAPI (blue), and imaged to determine cell size. Left panel, quantification; right panel, representative images. Means + SEM, n = 3. ns, not significant, * *p* < 0.05. Scale bar = 150 µm. (I) *NDC80* and *KIF23* mRNA levels in CAL-33 and MSK‑Leuk1 cells were determined by qRT‑PCR and normalized to those of *β‑2‑microglobulin* and of CAL‑33 cells using the ΔΔC_T_ method. Means + SEM, n = 3.

**Supplementary Figures S1 to S7**


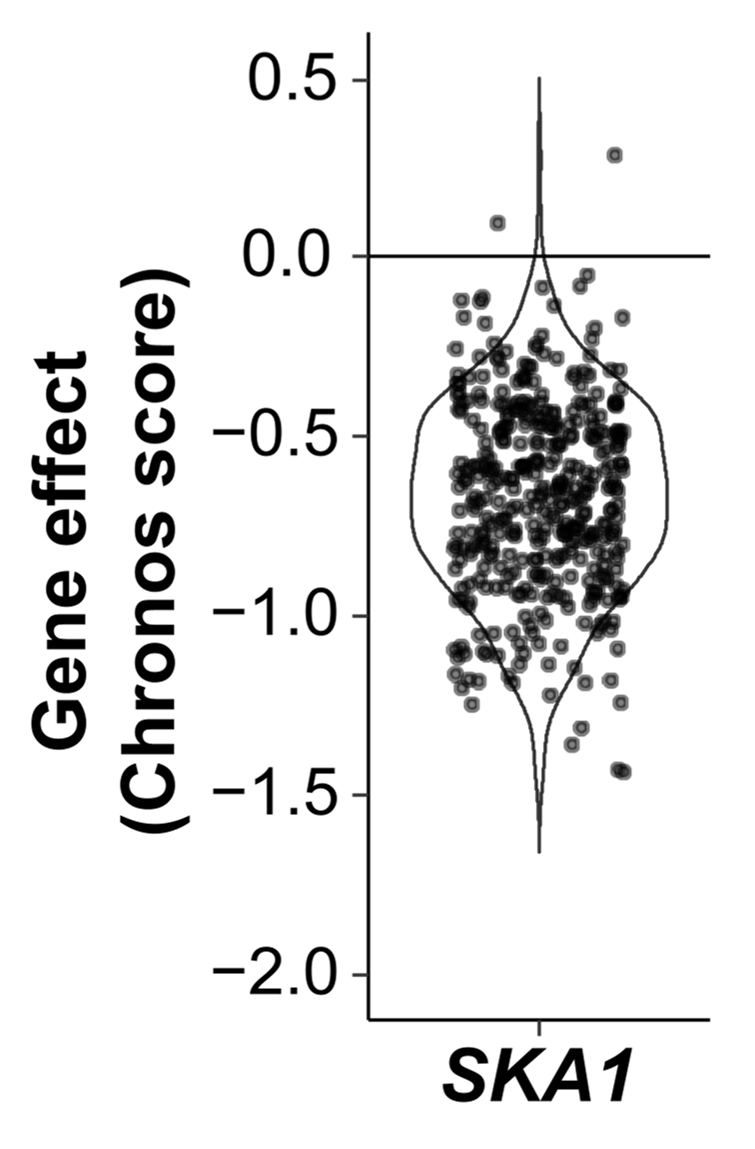


**Supplementary Figure S1**


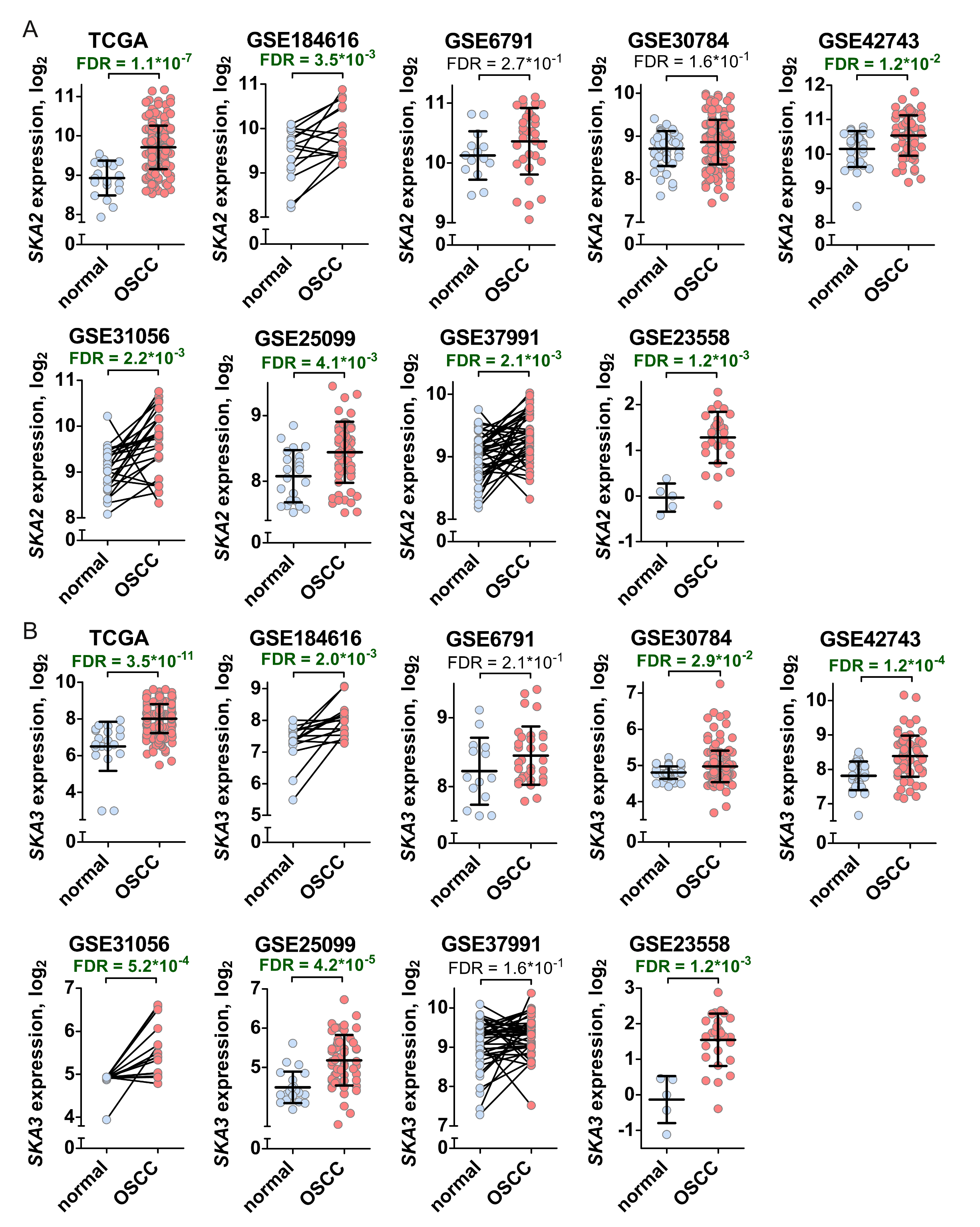


**Supplementary Figure S2**


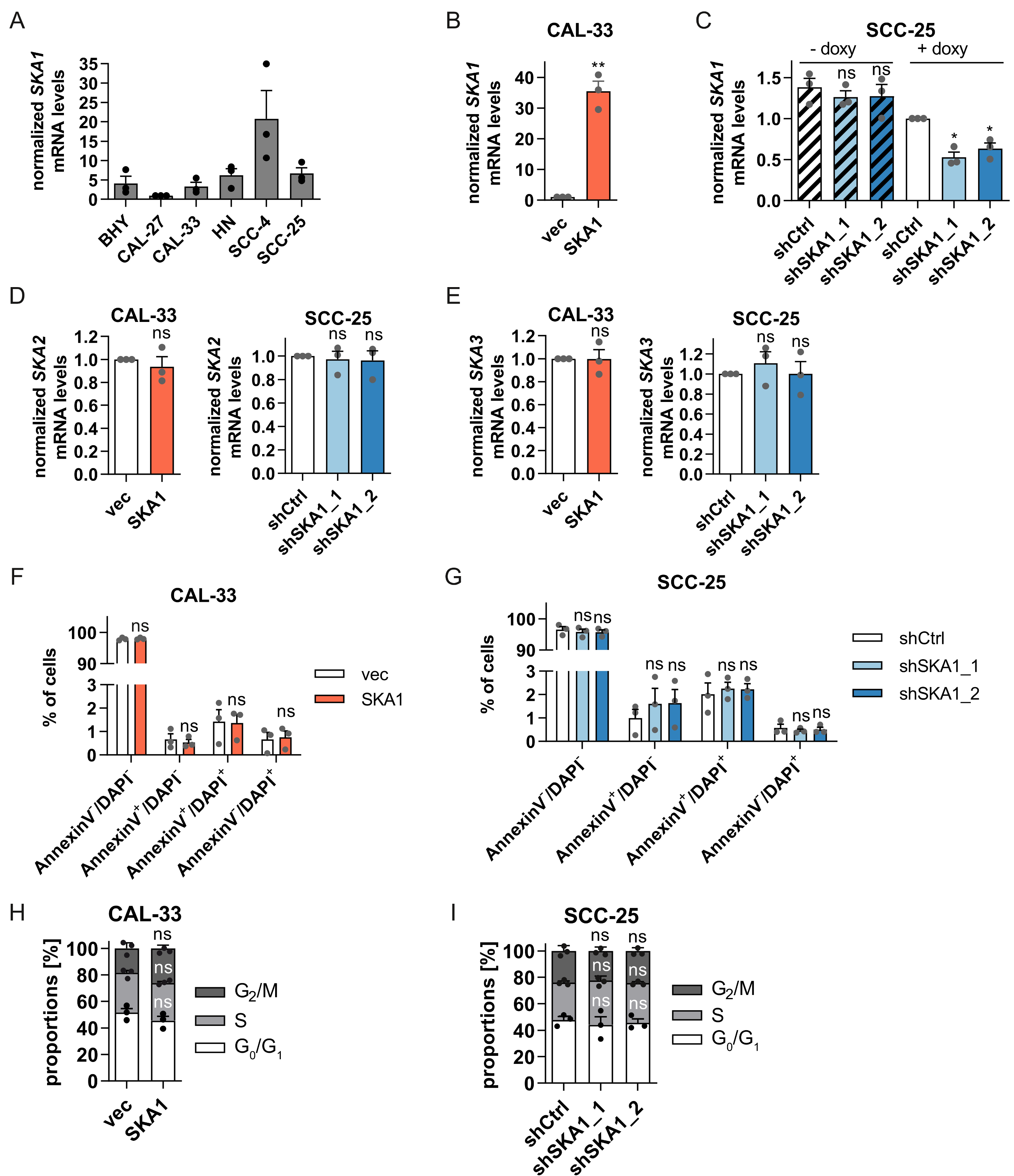


**Supplementary Figure S3**


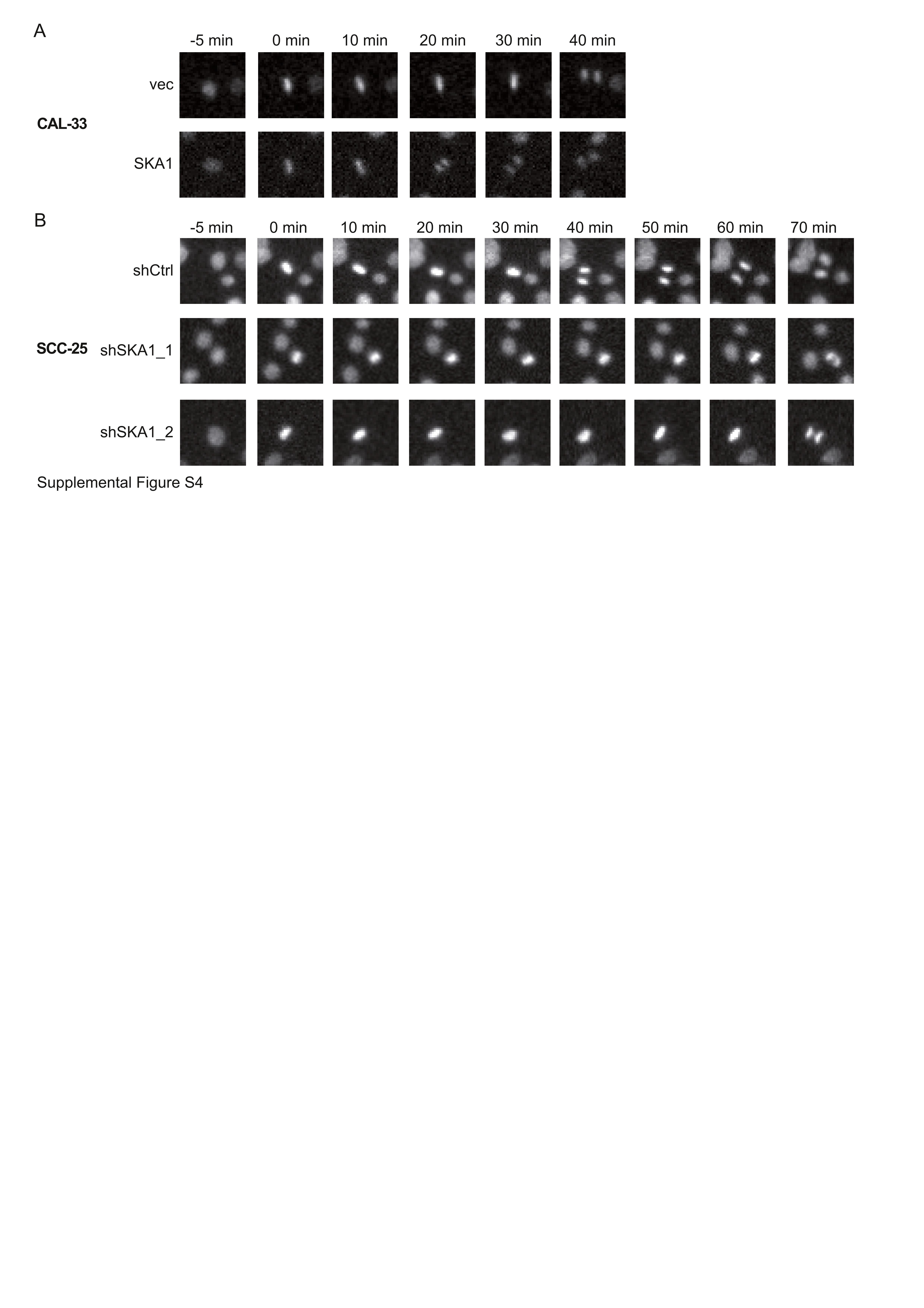


**Supplementary Figure S4**


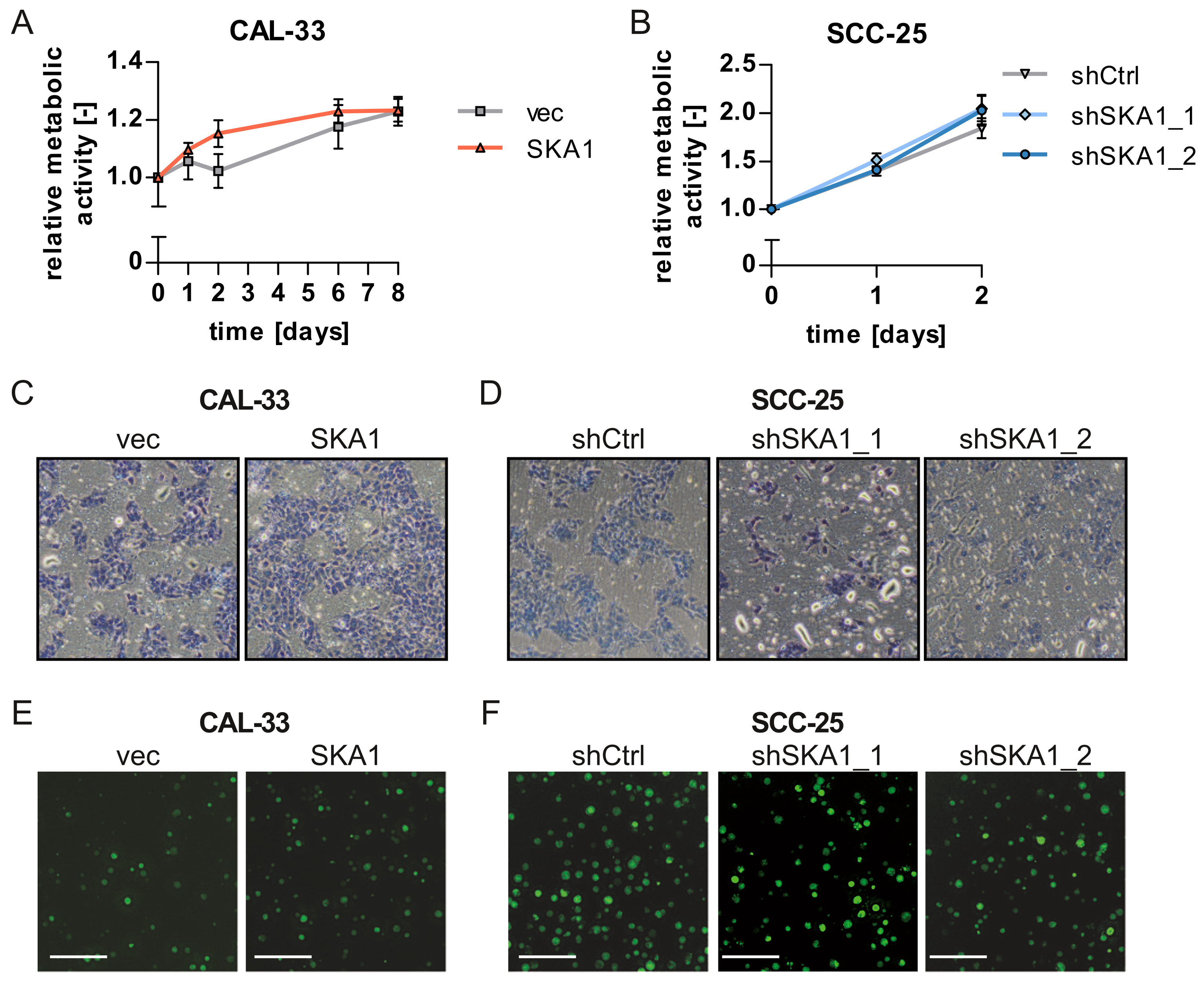


**Supplementary Figure S5**


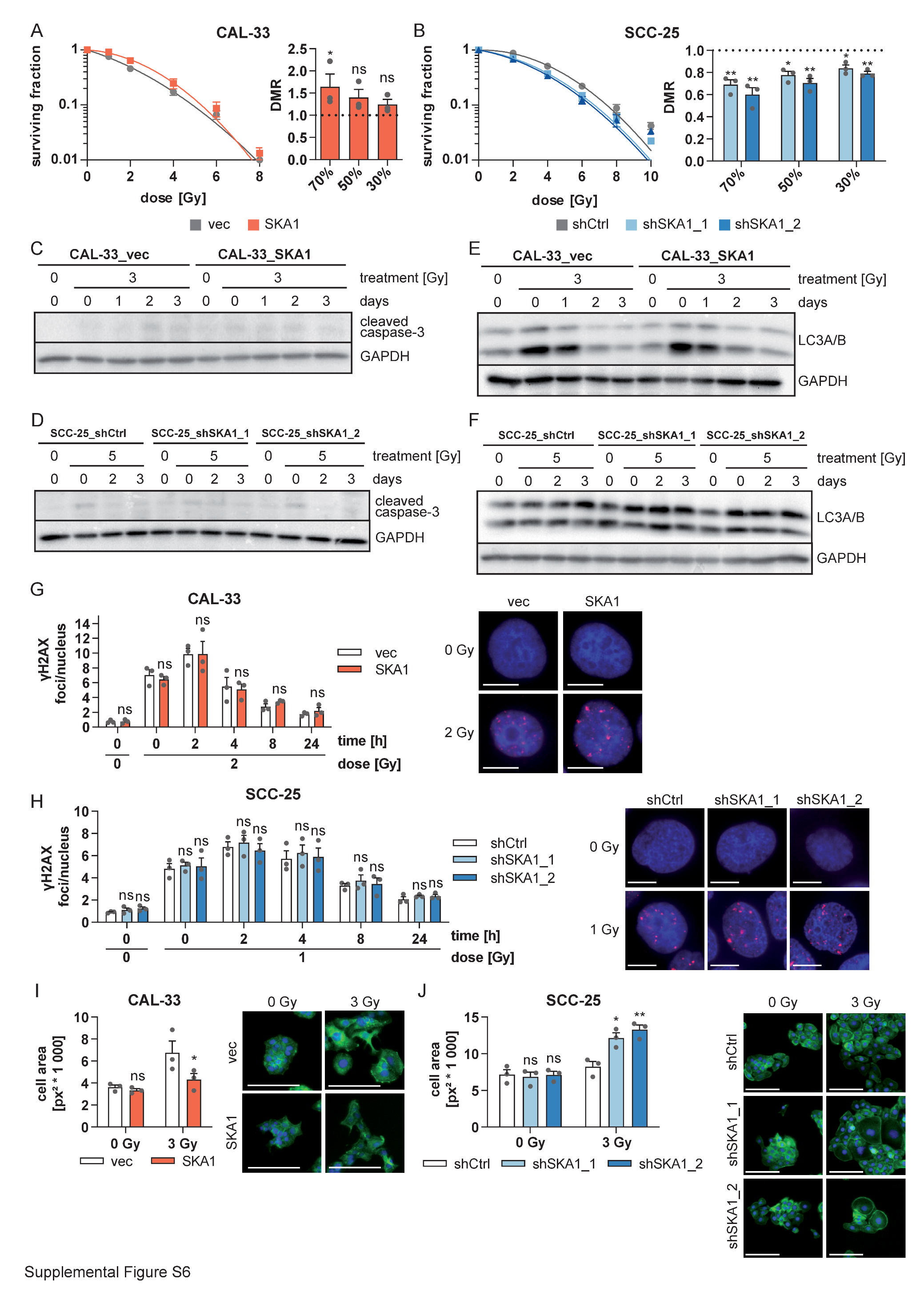


**Supplementary Figure S6**


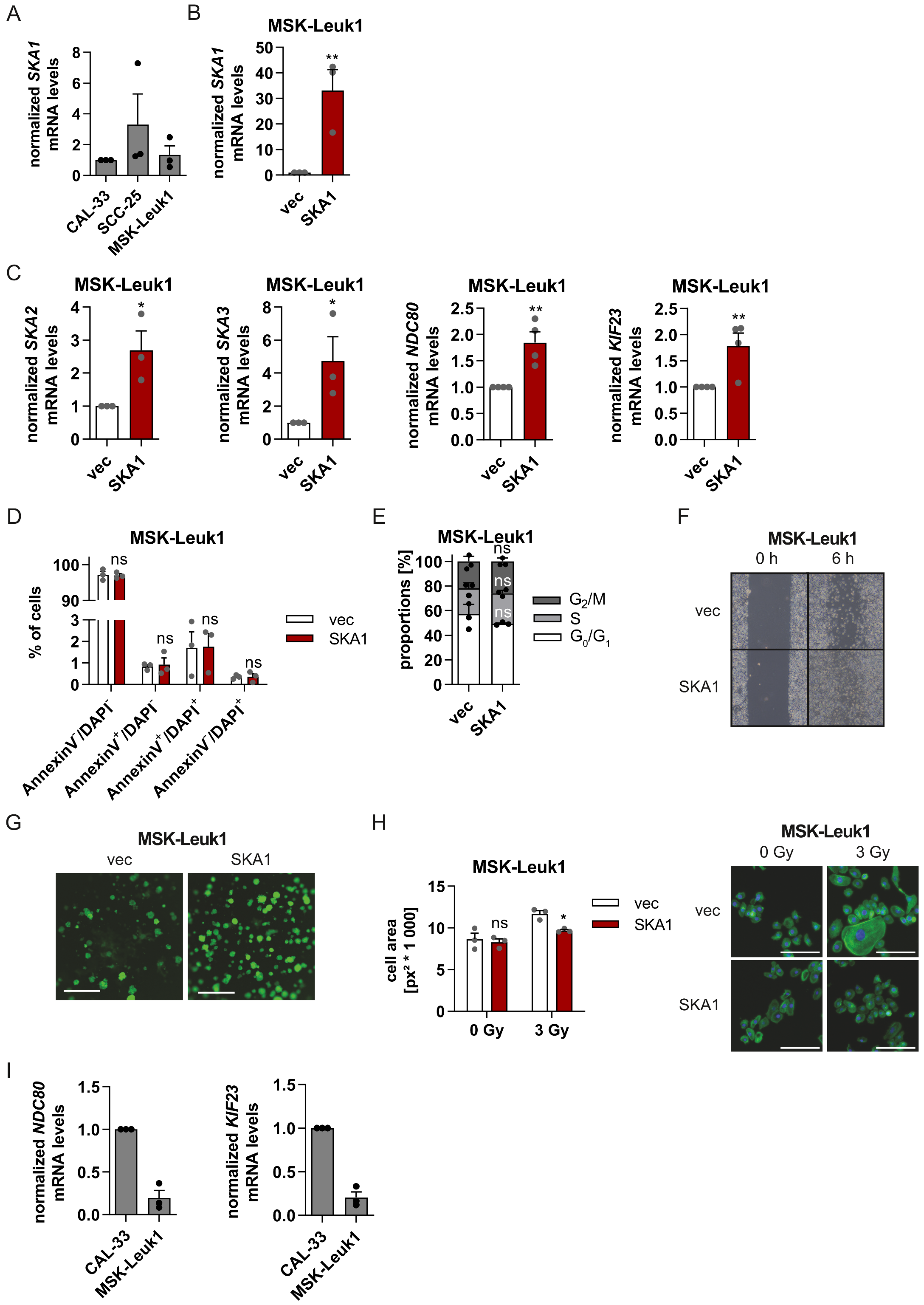


**Supplementary Figure S7**
